# Physical and insecticidal durability of Interceptor^®^, Interceptor^®^ G2, and PermaNet^®^ 3.0 insecticide-treated nets in Burkina Faso: results of durability monitoring in three sites from 2019 to 2022

**DOI:** 10.1186/s12936-024-04989-w

**Published:** 2024-06-04

**Authors:** Jacky Raharinjatovo, Roch Kounbobr Dabiré, Keith Esch, Dieudonné Diloma Soma, Aristide Hien, Tiecoura Camara, Mame Birame Diouf, Allison Belemvire, Lilia Gerberg, Taiwo Samson Awolola, Adama Koné, Djenam Jacob, Sophie Vandecandelaere, Marie Baes, Stephen Poyer

**Affiliations:** 1 PMI VectorLink Project, Population Services International, Antananarivo, Madagascar; 2 Institut de Recherche en Sciences de La Santé, Bobo-Dioulasso, Burkina Faso; 3 PMI VectorLink Project, Population Services International, Washington, DC USA; 4 Burkina Faso Permanent Secretariat for Malaria Elimination, Ouagadougou, Burkina Faso; 5 U.S. President’s Malaria Initiative, Ouagadougou, Burkina Faso; 6 U.S. President’s Malaria Initiative, USAID, Washington, DC USA; 7 U.S. President’s Malaria Initiative, Malaria Branch, US Centers for Disease Control and Prevention, Atlanta, GA USA; 8 PMI VectorLink Project, Abt Associates, Ouagadougou, Burkina Faso; 9 PMI VectorLink Project, Abt Associates, Washington, DC USA; 10 Centres Wallon de Recherches Agronomiques, Gembloux, Belgium

**Keywords:** Insecticide treated net, Physical durability, Piperonyl butoxide-synergist, Chlorfenapyr, Dual active ingredient, Insecticidal effectiveness, Burkina Faso

## Abstract

**Background:**

National Malaria Programmes (NMPs) monitor the durability of insecticide-treated nets (ITNs) to inform procurement and replacement decisions. This is crucial for new dual active ingredients (AI) ITNs, for which less data is available. Pyrethroid-only ITN (Interceptor^®^) and dual AI (Interceptor® G2, and PermaNet^®^ 3.0) ITNs were assessed across three health districts over 36 months in southern Burkina Faso to estimate median ITN survival, insecticidal efficacy, and to identify factors contributing to field ITN longevity.

**Methods:**

Durability was monitored through a prospective study of a cohort of nets distributed during the 2019 mass campaign. Three health districts were selected for their similar pyrethroid-resistance, environmental, epidemiological, and population profiles. Households were recruited after the mass campaign, with annual household questionnaire follow-ups over three years. Each round, ITNs were withdrawn for bioassays and chemical residue testing. Key measures were the percentage of cohort ITNs in serviceable condition, insecticidal effectiveness, and chemical residue content against target dose. Cox proportional hazard models were used to identify determinants influencing ITN survival.

**Results:**

At endline, the median useful life was 3.2 (95% CI 2.5–4.0) years for PermaNet^®^ 3.0 ITNs in Orodara, 2.6 (95% CI 1.9–3.2) years for Interceptor® G2 ITNs in Banfora and 2.4 (95% CI 1.9–2.9) years for Interceptor® ITNs in Gaoua. Factors associated with ITN survival included cohort ITNs from Orodara (adjusted hazard ratio (aHR) = 0.58, p = 0.026), households seeing less rodents (aHR = 0.66, p = 0.005), female-headed households (aHR = 0.66, p = 0.044), exposure to social behavior change (SBC) messages (aHR = 0.52, ≤ 0.001) and folding nets when not in use (aHR = 0.47, p < 0.001). At endline, PermaNet® 3.0 ITN recorded 24-h mortality of 26% against resistant mosquitos on roof panels, with an 84% reduction in PBO content. Interceptor^®^ G2 ITN 72-h mortality was 51%, with a 67% reduction in chlorfenapyr content. Interceptor^®^ ITN 24-h mortality was 71%, with an 84% reduction in alpha-cypermethrin content.

**Conclusion:**

Only PermaNet^®^ 3.0 ITNs surpassed the standard three-year survival threshold. Identified protective factors should inform SBC messaging. Significant decreases in chemical content and resulting impact on bioefficacy warrant more research in other countries to better understand dual AI ITN insecticidal performance.

**Supplementary Information:**

The online version contains supplementary material available at 10.1186/s12936-024-04989-w.

## Background

The global malaria community began intensifying its insecticide-treated net (ITN) scale-up in recent years. Population access to ITNs in sub-Saharan Africa was estimated to be just 3% in 2004 and increased to 54% in 2021, a period during which malaria mortality in sub-Saharan Africa decreased from an estimated 754,000 to 593,000 [1]. Measuring the durability and average useful life of ITNs helps to highlight changes in ITN coverage post-distribution [2]. Durability is measured as the length of time ITNs remain in a household effectively protecting users against malaria-transmitting mosquitoes, both as a physical barrier and through insecticide effectiveness.

In 2011, the World Health Organization (WHO) released standard guidance for countries to routinely monitor the durability of ITNs distributed following mass campaigns [3, 4]. This guidance facilitated National Malaria Programmes (NMPs) to estimate the average useful life of distributed nets and provided valuable post-market monitoring data for stakeholders interested in ensuring that ITNs were providing protection in line with their assumed three-year life span under field conditions. Many NMPs in sub-Saharan Africa have adopted the practice of routine durability monitoring, with support from donors including the United States President’s Malaria Initiative (PMI). Durability monitoring results from several countries are available for pyrethroid-only ITNs [5–12]. However, there are fewer published studies on dual active ingredient (AI) ITN field performance [13–16].

The emergence and escalation of pyrethroid resistance in key mosquito populations across sub-Saharan Africa has led to the introduction of new types of ITNs, which incorporate a second AI to complement the pyrethroid to kill, inhibit feeding, or reduce fertility in resistant mosquito populations. In 2017, the WHO formally recommended the use of piperonyl butoxide (PBO) synergist ITNs [17]. In 2023, new guidance was released which expanded WHO recommendations to include chlorfenapyr and pyriproxyfen ITNs, along with PBO ITNs for the prevention of malaria in adults and children in areas where mosquitoes have become resistant to pyrethroids [18]. These recommendations were released along with prioritization guidance for NMPs when procuring ITNs with limited resources [19]. In 2021, manufacturers delivered approximately 208 million ITNs to sub-Saharan Africa, of which approximately 113 million (55%) were dual AI ITNs (ITNs labelled as "PBO” and “dual a.i.” were separate in the source text calculation but combined and labelled as “dual a.i. ITNs” here.) [1].

In 2021, 3.3% of global malaria cases and 3.4% of global malaria deaths occurred in Burkina Faso (1). The absolute burden of malaria in Burkina Faso has remained largely unchanged in the past decade, with malaria cases decreasing by just 10% between 2011 (9.2 million) and 2021 (8.3 million). Over the same period, malaria deaths decreased by an estimated 46%, from 34,912 to 18,976 [1]. Between 2014 and 2018, household ITN ownership decreased from 91 to 76% in rural areas of Burkina Faso, a predominantly rural country [20, 21]. The ITN use:access ratio, which measures population level use in relation to population-level access, was classified as “good” (≥ 0.8) in nine regions, “below target” (≥ 0.6– < 0.8) in three regions and “poor” (< 0.6) in one region in 2018 [22]. Burkina Faso is also threatened by increasing pyrethroid resistance in prevailing malaria vectors [23–27]. Though no recent durability monitoring studies following standard methodologies have taken place in Burkina Faso, a 2017 evaluation demonstrated that unwashed Interceptor^®^ G2 ITNs restored mortality to 78% in pyrethroid-resistant mosquitoes, compared to 17% mortality against unwashed alpha-cypermethrin-only ITNs [28].

Unitaid and The Global Fund to Fight AIDS, Tuberculosis and Malaria, supported the Permanent Secretariat for Malaria Elimination (SP-Palu) to conduct a 2019 mass campaign in Burkina Faso through the provision of Interceptor® G2 and PermaNet^®^ 3.0 ITNs for distribution in the country’s southwest region. The SP-Palu targeted the distribution of these new nets in the southwest, where malaria prevalence was highest and pyrethroid resistance has been detected higher in local malaria vectors. The New Nets Project (NNP) collected surveillance data on vector bionomics, malaria epidemiology and human behaviors, which has contributed to the evidence base determining the public health value of dual AI ITNs (28).

Given their novelty and limited deployment to date, little durability monitoring data on multi-year field performance is published on dual AI ITNs [29, 30]. The field performance of new net types under natural conditions was studied for 36 months after the 2019 mass campaign in Burkina Faso. The study had two primary objectives. First, to assess the physical and insecticidal durability of Interceptor^®^, Interceptor^®^ G2, and PermaNet^®^ 3.0 ITNs in three nearby study sites over three years and estimate median ITN survival. Second, to compare physical durability results between the three locations with similar environmental, epidemiological and population profiles to identify significant socio-cultural behavioral determinants of ITN field performance.

## Methods

### Study sites and ITN brands

**Fig. 1:**
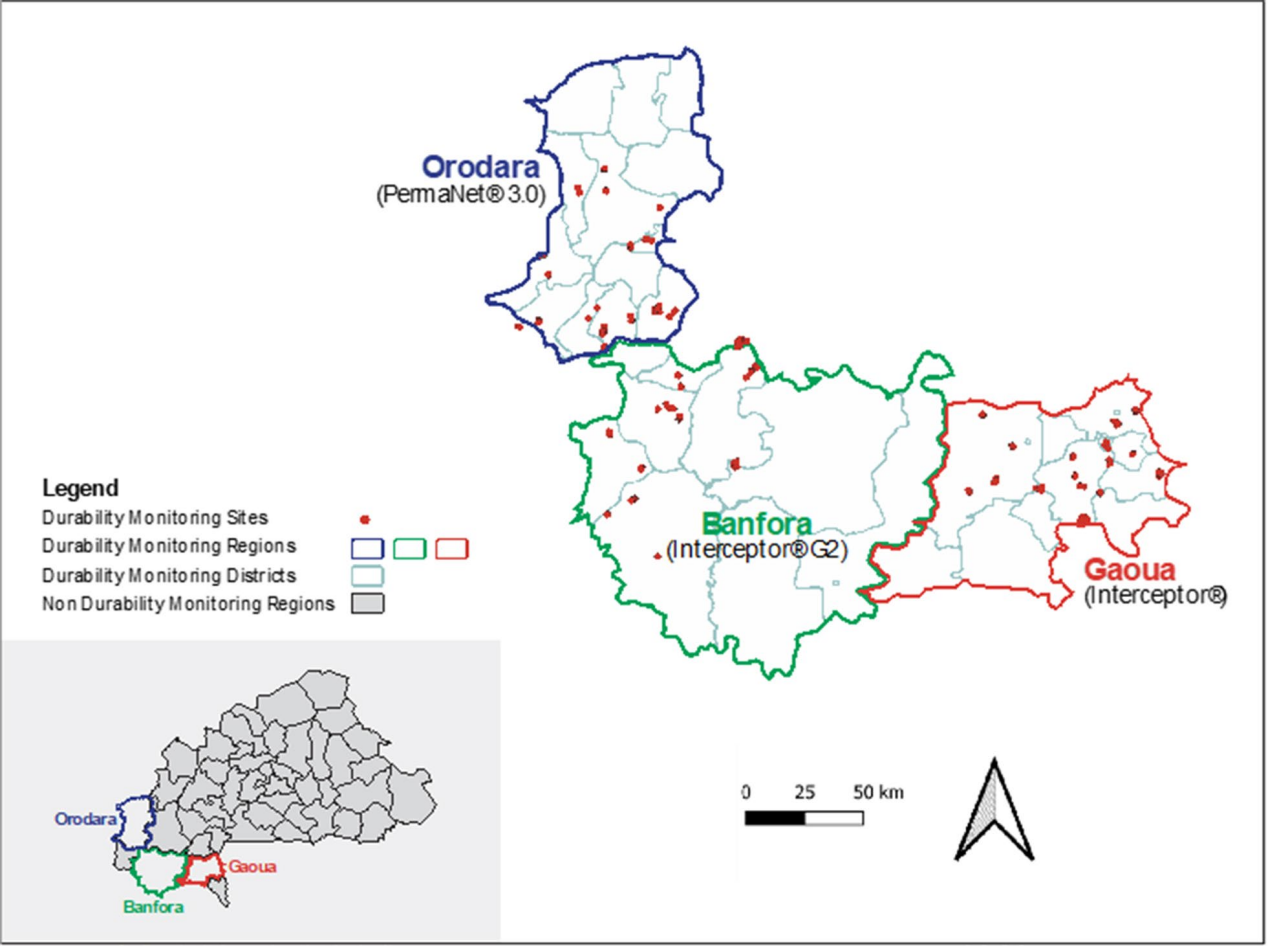
Locations of the Burkina Faso 2019–2022 ITNs Durability Monitoring of Interceptor^®^G2, Interceptor^®^ and PermaNet^®^ 3.0

This study was conducted in three districts in Burkina Faso, each of which received different ITNs as part of the 2019 mass ITN distribution campaign. Banfora health district in Comoé province received Interceptor^®^ G2 ITN, Gaoua health district in Poni province received Interceptor^®^ ITNs, and Orodara health district in Kénédougou province received PermaNet^®^ 3.0 ITNs (Fig. 1). Interceptor^®^ G2 ITNs were prioritized for distribution in the Southwest Region by the NMP due to the documented pyrethroid resistance in that area [24, 31] and high malaria prevalence rates [21]. Study sites were selected in collaboration with SP-Palu and PMI based on the location of entomological and epidemiological study sites for the NNP to ensure durability monitoring data would complement NNP studies. Additionally, durability monitoring study sites had similar environmental, epidemiological, and population profiles. The three study health districts experience hyperendemic malaria transmission and are in the west and southwest of Burkina Faso, where the rainy season lasts from May to October, leading to higher malaria transmission between May and October. Agriculture is the primary economic activity. The Annuaire Statistique 2017 reported malaria incidence per 1,000 people to be 729 in Banfora, 722 in Gaoua, and 631 in Orodara. According to the 2017–18 Malaria Indicator Survey, rates of ITN ownership, access, and use were highest in Banfora’s region (Cascades), followed by Orodara (Hauts-Bassins), and then Gaoua (Sud-Ouest) [21]. The 2018 ITN use: access ratio is classified as “good” in Cascades and Orodara, but “below target” in Sud-Ouest [22].

### Study design

This was a prospective cohort study of a representative sample of Interceptor^®^ G2, Interceptor^®^ and PermaNet^®^ 3.0 ITNs distributed as part of the 2019 mass ITN distribution campaign and followed up from 2019 to 2022 in three districts of Burkina Faso. The study design adhered to ITN durability monitoring guidance developed by PMI [32].

**Fig. 2:**
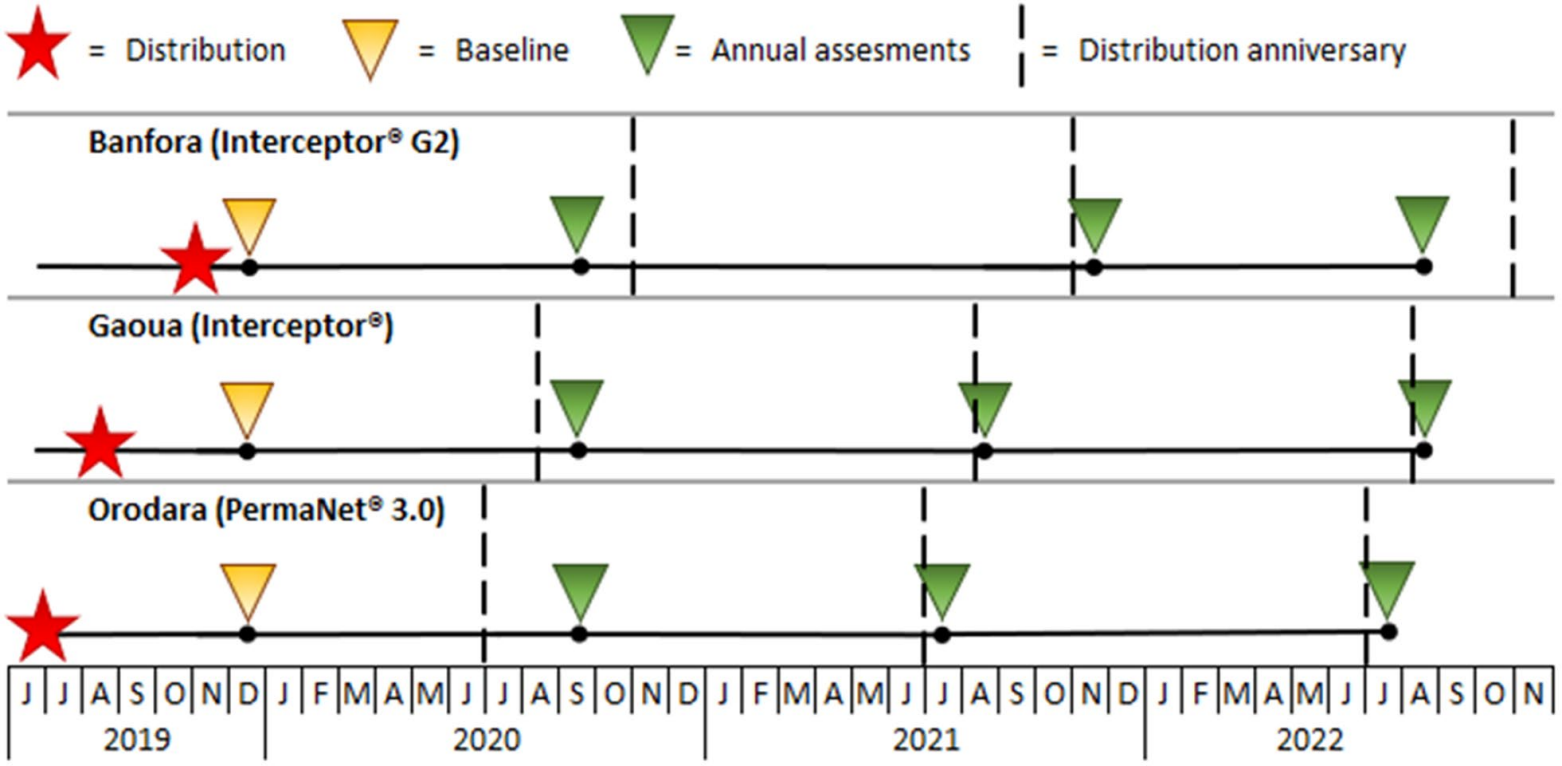
Durability monitoring timeline

The mass distribution campaign took place in three phases between June and November of 2019. PermaNet^®^ 3.0 ITNs (side: 100-denier, polyester, 2.1 g/kg deltamethrin; roof: 100-denier, polyethylene, 4.0 g/kg deltamethrin and 25.0 g/kg PBO) were distributed in Orodara in June and July. Standard Interceptor^®^ ITNs (100-denier, polyester, 5.0 g/kg alpha-cypermethrin) were distributed in Gaoua in August while Interceptor^®^ G2 ITNs (100-denier, polyester, 2.4 g/kg alpha-cypermethrin and 4.8 g/kg chlorfenapyr) were distributed in Banfora between October and November. Baseline data collection was conducted from December 9–20, 2019 (between one and six-months post-mass ITN distribution). Data collection for the 12-month study round was conducted from August 31–November 12, 2020; the 24-month study round was conducted July 4–November 8, 2021 (July 4–11 in Orodara, August 4–14 in Gaoua, November 2–8 in Banfora), and the 36-month study round was conducted July 4–August 19, 2022 (Fig. 2). For the 36-month survey, data collection in Banfora was conducted 33 months post-campaign in order to complete data collection prior to a mass distribution campaign that was scheduled to begin in the third quarter of 2022.

Household questionnaires were administered during each survey round to obtain information on cohort and non-cohort ITN status and potential risk factors for ITN durability. A standard hole assessment tool was used to assess the physical integrity of cohort ITNs. Additionally, data was collected on attritted cohort ITNs that were no longer present in the household at the time of the survey. Each round, 30 ITNs confirmed to have been distributed as part of the 2019 campaign (verified by their label and owner recall) were withdrawn from each site to undergo insecticidal effectiveness testing. Withdrawn ITNs were replaced on a like-for-like basis with new ITNs of the same brand. Cone bioassays, tunnel tests and chemical content testing were performed at all rounds.

### Sample size and sampling

Sample size calculations were made assuming a design effect of 2.0, household loss to follow-up of 5% over three years, two-sided alpha error of 0.05, and 80% power [33]. An initial sample of 150 households per location was created to ensure an initial cohort of 525 nets per location, or 1,575 nets from the 450 households in the three locations. During monitoring of the baseline survey round, the mean household size was smaller than anticipated leading to fewer ITNs per household. Two clusters were added to the sample in each site to ensure a sufficient sample size to power the study. This sample size allowed for the detection of a 6.7 percentage-point difference between any two locations with an assumed median survival of 3 years: e.g., 43% or less or 57% or more estimated survival compared to 50%. This translated into approximately a 0.5 median survival difference that was able to be detected as statistically significant.

Households were selected using two-stage sampling. At the first stage, 15 clusters (then increased to 17) were sampled per study district. A cluster was defined as the lowest level geographic areas used for the campaign planning. The selection of 17 clusters in each region proceeded with probability proportionate to size, with the number of ITNs distributed per cluster as the measure of size. Cluster household lists were used to select 10 households per cluster, using simple random sampling at the second stage. In each randomly selected household, ITNs that were part of the 2019 mass campaign were identified and enrolled into the study.

### Training and fieldwork procedures

Fieldwork was conducted in each study site by implementation teams of nine people, staffed by Institut de Recherche en Sciences de la Santé (IRSS). Each implementation team included a coordinator and two sub-teams of one supervisor and three interviewers each. Staff were carefully selected based on their knowledge of the local language and experience conducting household surveys. At baseline, a training of trainers (TOT) was led by a PMI VectorLink staff member experienced in durability monitoring with support from the SP-Palu. Baseline training was then provided to the data collection team by IRSS. Refresher TOTs for the 12-, 24- and 36-month rounds were conducted online by PMI VectorLink staff due to COVID-19-related travel restrictions. Subsequent in-person data collection team training was conducted by IRSS with support by SP-Palu. The training covered: study design and sampling procedures, ethical considerations (such as consent), COVID-19 adaptations, a detailed review of the questionnaire with role play, the use of tablets and the SurveyCTO software, and the physical assessment of holes and net repairs with practical exercises. Upon entering a cluster, data collection teams engaged with local authorities to gain approval to collect data, describe study objectives and answer questions. In most clusters, local community members served as guides to assist study teams to locate study households during follow-up rounds. Standard COVID-19 safety measures were followed throughout the study. No households were screened out of the survey due to COVID-19 risks.

### Laboratory procedures

Susceptible and resistant mosquito strain rearing, characterization and bioassay testing was carried out in IRSS’s insectary and laboratory located in Ouagadougou, Burkina Faso. Bioassays were conducted in line with the Innovation to Impact SOPs [34, 35].At each survey round, standard WHO cone bioassays and tunnel tests were used to determine insecticidal effectiveness using insecticide-susceptible *Anopheles gambiae* Kisumu mosquitoes and pyrethroid-resistant *Anopheles coluzzii* VKPER strain [36]. Both strains were characterized at each survey round prior to performing the bioassays using WHO susceptibility test kits with 0.05% deltamethrin (the pyrethroid in PermaNet^®^ 3.0), and 0.05% alpha-cypermethrin (the pyrethroid in Interceptor^®^ and Interceptor^®^ G2). Tests were also carried out with deltamethrin 0.05% after pre-exposure to PBO 4% (the synergist in PermaNet^®^ 3.0).

Cone bioassays were used for Interceptor^®^ ITN brand. The susceptible mosquito strain (*An. gambiae* Kisumu) was used with five mosquitoes per cone, four panels tested from each net (three sides and the roof) and two replicates per panel (eight cone tests with 40 susceptible mosquitoes per net in total). Thresholds for optimal (60-min knock-down (KD60) ≥ 95% or mortality ≥ 80%) and minimal (KD60 ≥ 75% or mortality ≥ 50%) effectiveness were defined using KD60 and mortality results.

Cone bioassays were also used for PermaNet^®^ 3.0 ITN brand containing PBO. The susceptible (*An. gambiae* Kisumu) and resistant (*An. coluzzii* VKPER) mosquito strains were used with five mosquitoes per replicate, with two replicates per net piece (two pieces from side panels and two pieces from roof panel), with a total of eight cone tests with 40 susceptible and 40 resistant mosquitoes per net sample. In each case, the KD60 and the 24-h mortality were measured. Abbott’s formula was used to adjust mortality based on negative control results. Additionally, positive and negative control nets were tested along with the net samples withdrawn from the field. Three new control nets were tested per day: an untreated net, a new pyrethroid + PBO net, and a new pyrethroid-only net. The new untreated negative control net was tested against susceptible and resistant mosquito strains. The new PermaNet^®^ 3.0 and new pyrethroid-only ITNs as positive control were tested against resistant mosquito strain. For each of the three control nets, one piece was used with five mosquitoes per cone and two replicates (six cone tests with 30 mosquitoes per net per day in total). KD60 and 24-h mortality were measured.

Tunnel tests were used for Interceptor® G2 ITNs against insecticide-susceptible *An. gambiae* Kisumu and pyrethroid-resistant *An. coluzzii* VKPER mosquitoes. For each sampled net, two samples were taken from the net roof panel and two samples were taken from the net side panels for bioassays. Three control nets were used during testing: one untreated (negative) control, one new Interceptor^®^ net (alpha-cypermethrin only), and one new Interceptor^®^ G2 net. Tunnel tests were completed over 3 days [36], and so each control net had three replicates, with results shared among all pre-distribution Interceptor^®^ G2 nets tested on the same day. Between 80 and 120 pyrethroid-resistant female mosquitoes, aged five to eight days, and sugar-starved for 6 h, were introduced into the end opposite of the bait at 18:00. The lights in the room were turned off and only turned on when the tunnel test finished the following morning at 7:00, giving an exposure period of 12–15 h. At 7:00, mosquitoes were collected from the tunnel, noting the compartment in which the mosquitoes were collected (initial compartment/animal compartment), the blood feeding status (fed/unfed), and mortality (living/dead). Living mosquitoes were put into cups covered with untreated netting, and cotton wool soaked in sugar solution was placed on top of the cups, allowing mosquitoes to feed ad libitum [36]. Mortality was recorded at 18:00 (24 h after the tunnel test started), and then again at 48 and 72 h. Insecticidal effectiveness was measured by estimating 24- and 72-h mortality, net penetration, level of blood feeding, and BFI.

For each net, 30 cm × 30 cm squares were cut in areas adjacent to those made for bioassay testing and pooled to obtain a homogeneous and representative sample of the entire net [34, 35]. These samples were tested at the Centre Wallon de Recherches Agronomiques in Belgium using the standard Collaborative International Pesticides Analytical Council methods cited in the WHO specification for each ITN [37, 38]. Chemical tests measured pooled mean and median AI levels across the ITN samples in g/kg, compared with manufacturer specifications.

### Data management

Open Data Kit (ODK) software was employed on Android tablets to implement the household questionnaire. Logic checks and field constraints were built into the ODK tool to strengthen data quality and reduce data cleaning. Open Street Map for Android was used to geo-locate study households. Data was uploaded by data collection teams to a secure online server either daily, or when an internet connection became available. When data collection for each survey round was complete, data was moved into Stata (version 12) to be cleaned, managed, and analyzed. Longitudinal household- and cohort-ITN level data sets were produced by combining cross-sectional data from each round to facilitate ITN survival analysis.

### Definition of outcomes

The primary study outcome was physical ITN survival, defined as the proportion of cohort ITNs still present and in serviceable condition. This outcome combines measures of ITN attrition and ITN physical integrity. ITN attrition describes cohort nets no longer present in a study household. There are two categories of attrition: known and unknown outcomes. Known outcomes are nets attritted due to wear and tear including nets thrown away, nets destroyed, or nets used for other purposes. Unknown outcomes include nets given to relatives, nets given to others, nets that were stolen or sold, and nets that were reported as lost for unknown reason. Nets with unknown outcomes were excluded from the estimation of ITN survival.

ITN physical integrity was measured using the WHO proportionate Hole Index (pHI) [3]. During the hole assessment, the number of holes in each panel of the ITN were counted, using four different size categories (size 1: 0.5–2 cm, size 2: 2–10 cm, size 3: 10–25 cm and size 4: larger than 25 cm in diameter). Weights were applied to the count of the number of holes in each size category and the weighted sum was then used to categorize each cohort ITN as “good”, “damaged”, “torn” and “serviceable” [3]:

Good:total hole surface area < 0.01 m^2^ or pHI ≤ 64.

Damaged:total hole surface area 0.01–0.1 m^2^ or 64 < pHI ≤ 642.

Torn:total hole surface area > 0.1 m^2^ or pHI > 642.

Serviceable:total hole surface areas ≤ 0.1 m^2^ or pHI ≤ 642.

Median survival was calculated using two methods. At each round, the proportion of cohort ITNs surviving in serviceable condition was plotted against hypothetical survival curves with defined median survival values, and the median survival was taken as the relative position of the data point on a horizontal line between the two adjacent median survival curves. After the final survey, median survival was calculated as the linear extrapolation of the survival values from the last two rounds to the y = 50% line, provided both points were below 85% (when the hypothetical survival curves are themselves approximately linear) using the following formula:

$${\text{tm}} = {\text{t1 }} + \, \left( {{\text{t2 }}{-}{\text{ t1}}} \right) * \left( {{\text{p1 }}{-}{ 5}0} \right) \, / \, \left( {{\text{p1 }}{-}{\text{ p2}}} \right)$$

where tm is the median survival time, t1 and t2 the first and second time points in years and p1 and p2 the proportion surviving to first and second time point respectively in percent. Confidence intervals (CI) for this estimate were calculated by projecting the 95% CI from the survival estimates in the same way as described above.

### Definition of explanatory variables

The definitions of the explanatory variables and the analytic approaches were aligned to those from recently published literature [5, 6, 9, 10], that permitted to encourage and facilitate comparative review of results from completed durability monitoring studies. Explanatory variables were measured at the household- and cohort ITN-levels and represented risk factors for ITN damage. Risk factor groupings included respondent-level factors such as exposure to ITN messaging and attitudes to ITN care and repair; cohort ITN sleeping space environment factors, such as the type of sleeping space and whether cooking or food storage occurs in the same physical space; and cohort ITN handling factors, such as folding or tying up ITNs during the day and how ITNs are washed and dried.

Household attitudes towards nets and care and repair were measured using Likert scale questions. Statements were read to the respondent and the level of agreement was recorded. The four-level Likert scale was recoded to have a value of − 2 for “strongly disagree”, − 1 for “disagree”, + 1 for “agree” and + 2 for “strongly agree.” The attitude scores for each respondent were added together and divided by the number of statements to calculate an overall attitude score. A negative score represents a negative attitude, a score of 0 represents a neutral attitude, and a positive score represents a positive attitude. Attitude scores for net use and for net care and repair were calculated, as well as the proportion of households with a score above 1 (very positive attitudes) for each of the two attitude scores.

Responses to questions were aggregated across all rounds in which a cohort ITN or household participated. Responses were categorized as “never” if the response was “no” or “never” for all rounds; “at times” if the response was “sometimes” in at least one survey round or conflicting responses were recorded; “always” if the response was “yes” or “always” for all surveys.

### Statistical analyses

Chi-squared tests and contingency tables were used to compare proportions. Chi-squared tests for trends were employed to identify differences in proportions where more than two groups were being compared. Reported p-values for comparisons across study sites use this test for trend; no pairwise comparisons were performed. Medians and means were used to analyze continuous variables, and the one-way analysis-of-variance (ANOVA) was used to compare results between more than two groups for non-parametric and normally distributed data, respectively. Standard errors and 95% CI were calculated, as is appropriate for the clustered survey design. Survival analysis was calculated with risk of failure beginning at date of distribution, and separately, with risk of failure beginning when the net was first observed or reported hanging. ITN failure was defined when it attritted due to wear and tear (ITN was thrown away, destroyed, or used for other purposes) or its physical integrity classification was categorized as “torn.” Respondent recall was used to calculate time to failure based on the duration that the net was in the possession of the household. The midpoint between the previous two survey rounds was used as the failure time in cases where the respondent could not recall time to failure, or the net’s physical integrity failed. Cox proportional hazard models were created to identify determinants of ITN survival. Robust standard errors were employed during analysis to address net clustering within eligible households. Two Cox proportional hazard models were fit following the two survival analysis approaches: a household-level model fit failures with risk, starting at the distribution date and considering household-level determinants that were invariant for nets within a given household (e.g. study district, household size, household head characteristics, household wealth tertial); and a net-level model fit failures with risk starting the on date of the first observation that the net was hanging and considering the household-level determinants above and net-level determinants such as handling and types of user. For both approaches, univariate analysis identified factors significantly associated with survival, and all significant variables at the p = 0.2 level were included in a first multivariate model. Models were then refined by dropping non-significant variables (at the p = 0.1 level) and comparing model specifications using the likelihood-ratio test. Schoenfeld residual plots and analysis of time-varying covariates were used to check the proportionate hazard assumption. For the net-level model, risk of failure started on the date the net was first hung. Additional factors used in the net-level model included whether the net was folded up when not in use, type of sleeping space, whether the net was ever washed, whether the net was dried on bushes, and whether detergent was used for net washing. Washing was omitted in the final model as it was highly correlated with drying outside and using detergent for washing.

## Results

### Household cohort ITN sample description

At baseline, an adjustment was made to the number of clusters included in each study arm due to an underestimated mean household size assumption in the sample size calculation. Rather than the originally planned 15 clusters district, 17 clusters were visited per site each round. Despite the increased number of clusters visited per site, only 923 cohort ITNs were enrolled, which was less than the anticipated 1557 cohort ITNs needed to detect a 6.7 percentage-point difference between sites in median survival time. Ultimately, the final sample of 923 cohort ITNs allowed for the detection of a difference of 8.8 percentage-points in median ITN survival across study sites.

Table S1 presents the final status at endline of 510 study households containing 923 cohort ITNs. Of the 510 households, 61% (103/170) still had one or more cohort nets in Orodara, followed by 45% (77/170) in Gaoua, and 29% (50/170) in Banfora. A higher percentage (41%) of households in Gaoua have lost all their cohort nets compared to Banfora (32% (54/170)) or Orodara (19% (33/170)).

### Household characteristics

This section describes the general characteristics of the sampled households collected at the first survey round, including respondent demographic information, housing characteristics, and household assets as presented in Table S2. Household size was larger in Gaoua (4.26) and in Orodara (4.48) than in Banfora (3.44; p < 0.001). The proportion of female-headed households differed significantly across study districts (12% in Orodara, 18% in Banfora, and 29% in Gaoua; p = 0.004). In Gaoua and Orodara, 75% of male household heads were illiterate, compared to 54% in Banfora (p = 0.012). Illiterate women comprised the majority of female-headed households across districts (91% in Orodara, 89% in Gaoua, and 83% in Banfora). Across study sites, a higher proportion of dwellings had roofs made with finished materials in Orodara (96%) and in Banfora (92%) compared to Gaoua (79% p = 0.002). A higher percentage of households in Banfora (82%) contained flooring built with improved materials compared to Orodara (64%) and Gaoua (56%; p = 0.013). Use of charcoal for cooking was relatively uncommon across the three districts (14% in Banfora, 7% in Gaoua, and 2% in Orodara). In Gaoua, 74% of households reported ownership of livestock, compared to 63% in Banfora and 59% in Orodara. Chickens and goats were the households’ primary livestock. Radio ownership was more prevalent in Orodara (77%) and Banfora (70%) than in Gaoua (39%; p < 0.001).

### Risk factors for durability (household environment and ITNs environment)

Table S3 presents risk factors for ITN physical and chemical durability that were collected and monitored at each study timepoint. Lower proportions of households reported always storing food in a room used for sleeping in Banfora (15%) and Orodara (14%), compared to Gaoua (56%; p < 0.001). More households reported seeing rodents in each survey round in Gaoua (56%), followed by Orodara (48%) and Banfora (35%; p < 0.001). Almost no households in Banfora, Orodara, or Gaoua (< 1%) reported always cooking food in a room used for sleeping. However, this practice was reported as sometimes being done by 62% of households in Gaoua, 19% in Banfora, and 18% in Orodara (p < 0.001).

In Orodara, 52% of households reported having received SBC messages on net use and net care at least once across two separate survey rounds, higher than both Banfora (34%) and Gaoua (8%; p = 0.001). Nearly two thirds (62%) of households in Orodara reported positive net use attitudes in at least two survey rounds, compared to 42% in Banfora and 6% in Gaoua. (p < 0.001). Just 2% of households in Gaoua expressed positive net care attitudes in at least two survey rounds, compared to 27% in Banfora and 21% in Orodara (p < 0.001).

Cohort net durability was evaluated in relation to various aspects of net use and net care (Table S4). Across the four survey rounds, 89% of cohort nets were reported as being used in Orodara (89%), compared to Gaoua (84%), and 74% in Banfora (p = 0.016). In Orodara and Gaoua, a higher proportion of nets were reported as ever being hung, compared to Banfora (82% in Gaoua and 88% in Orodora higher than 69% in Banfora, p = 0.002). Orodara had the highest proportion of hanging nets never folded up (94%), followed by Banfora (51%) and Gaoua (22%; p < 0.001). Banfora reported the lowest percentage of nets that had ever been washed (51%), compared to 78% in Gaoua and 88% in Orodara (p < 0.001). A high proportion of nets were always dried outside after every washing reported across all survey rounds (97% in Banfora, 91% in Gaoua, and 81% in Orodara, p = 0.013). Drying nets on bushes or fences was uncommon across districts (11% in Gaoua, and only 3% in Banfora and Orodara, p = 0.004).

### Attrition

**Fig. 3:**
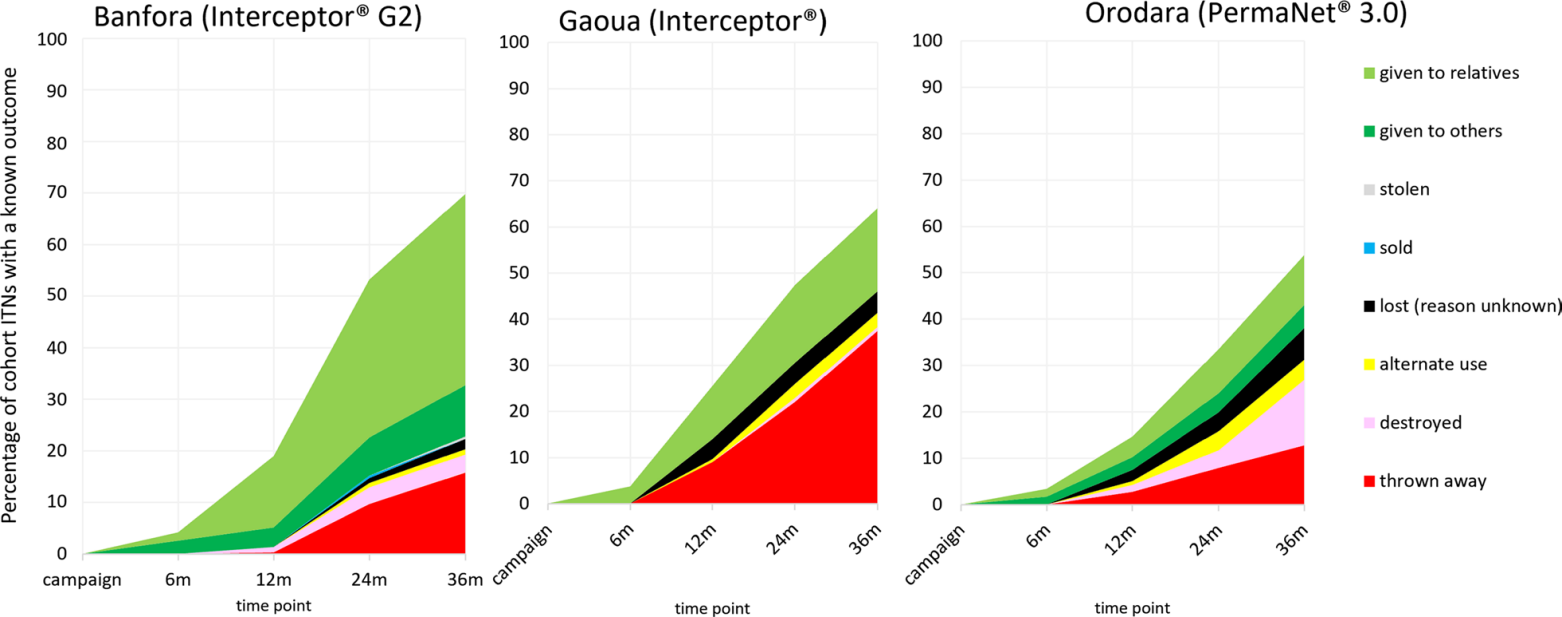
Reported causes of ITN attrition in the Burkina Faso 2019–2022 durability monitoring of interceptor^®^ G2, interceptor^®^, and PermaNet^®^ 3.0 study

At study endline, total ITN attrition was highest in Banfora (70%), followed by Gaoua (64%), and lowest in Orodara (54%, p = 0.066) (Fig. 3). At study endline, the primary cause of attrition was nets being given to relatives in Banfora (37%), nets thrown away in Gaoua (38%), and nets being destroyed in Orodara (14%). Attrition due to wear and tear, which is the combination of nets thrown away, destroyed, and repurposed for an alternate use, was highest in Gaoua (41%), lower in Orodara (31%), and lowest in Banfora (20%, p = 0.013). Tears were the most commonly reported damage mechanism across all districts and rounds (82% in Banfora, 54% in Gaoua, and 62% in Orodara, p = 0.204).

### Physical integrity

At study endline, Orodara had the highest proportion of nets in serviceable condition (90%), compared with Banfora and Gaoua (80% in both districts). In Banfora and Gaoua, the proportion of nets classified as ‘’too torn’’ increased over time, from 4 and 3%, respectively, at the second round to 20% in both districts by the study endline.

### Survival

**Fig. 4:**
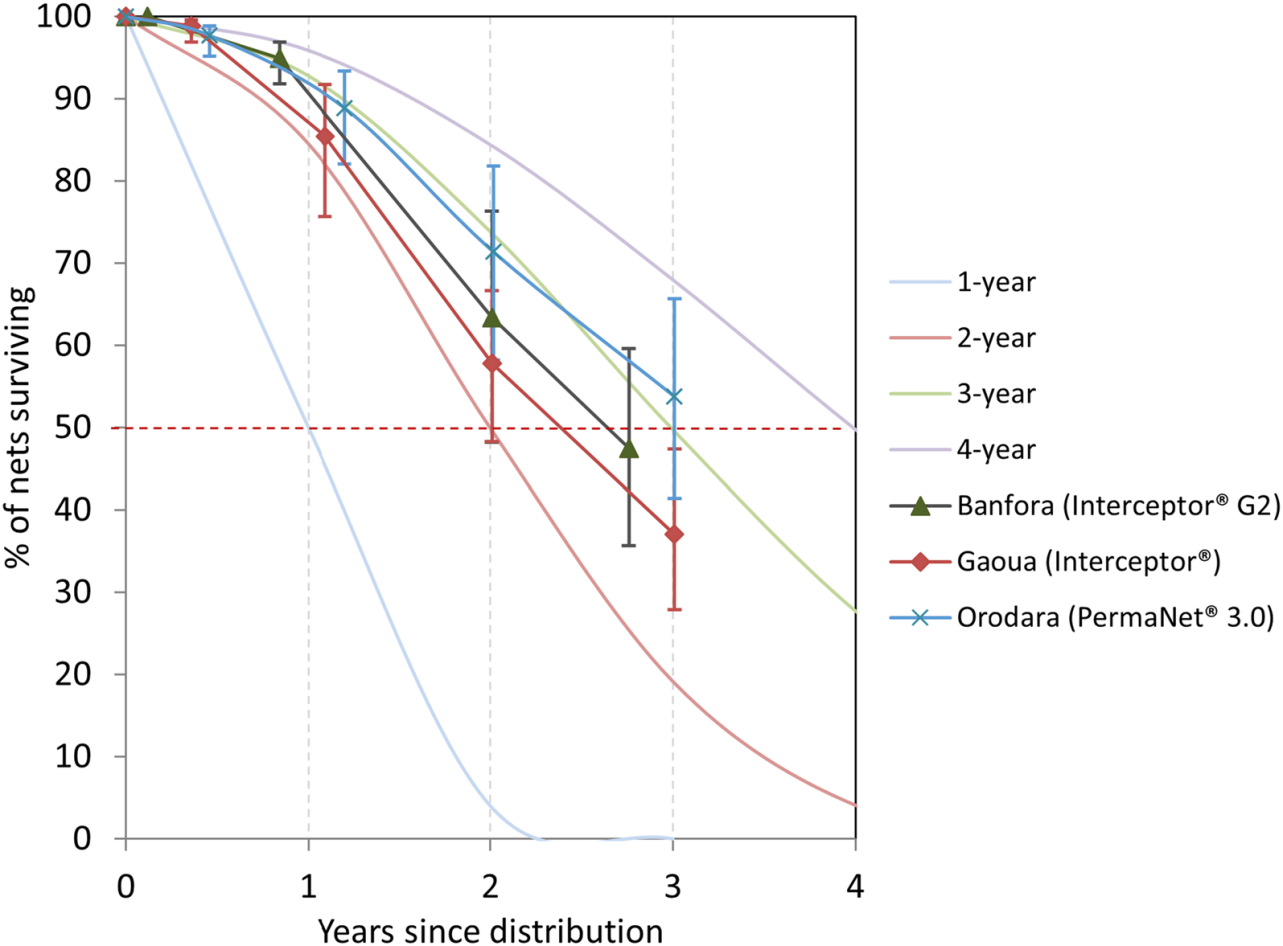
Physical Survival of Burkina Faso 2019–2022 Durability Monitoring of Interceptor^®^ G2, Interceptor^®^, and PermaNet^®^ 3.0 Study

The proportion of nets that survived in serviceable condition at study endline was highest in Orodara (54%), followed by Banfora (48%), and lowest in Gaoua (37%). Between the second survey round and study endline, the proportion of nets surviving in serviceable condition decreased by an average of 43% (Table 1). Using estimates from the last two survey rounds (24- and 36-months), the median useful life in years for Interceptor^®^ G2 in Banfora was 2.6 (95% CI 1.9–3.2) years, 2.4 (95% CI 1.9–2.9) years for Interceptor^®^ in Gaoua, and 3.2 (95% CI: 2.5–4.0) years for PermaNet^®^ 3.0 in Orodara (Fig. 4).

**Table 1:** Cohort ITNs surviving in serviceable condition and estimated median survival in years

	Second round % (95% CI)	Third round % (95% CI)	Study endline % (95% CI)
Banfora (Interceptor^®^ G2)			
Percentage surviving in serviceable condition	94.9 (91.8–96.9)	63.4 (48.1–76.3)	47.5 (35.6–59.7)
Median survival in years			
Graphical interpretation from Fig. 4		2.4	2.7
Calculated from last two data points			2.6 (1.9–3.2)
Gaoua (Interceptor^®^)			
Percentage surviving in serviceable condition	85.0 (75.4–91.3)	57.8 (48.2–66.7)	37.1 (27.9–47.4)
Median survival in years			
Graphical interpretation from Fig. 4		2.2	2.6
Calculated from last two data points			2.4 (1.9–2.9)
Orodara (PermaNet^®^ 3.0)			
Percentage surviving in serviceable condition	88.6 (81.7–93.1)	71.4 (58.1–81.8)	53.8 (41.4–65.8)
Median survival in years			
Graphical interpretation from Fig. 4		2.9	3.2
Calculated from last two data points			3.2 (2.5–4.0)

**Fig. 5:**
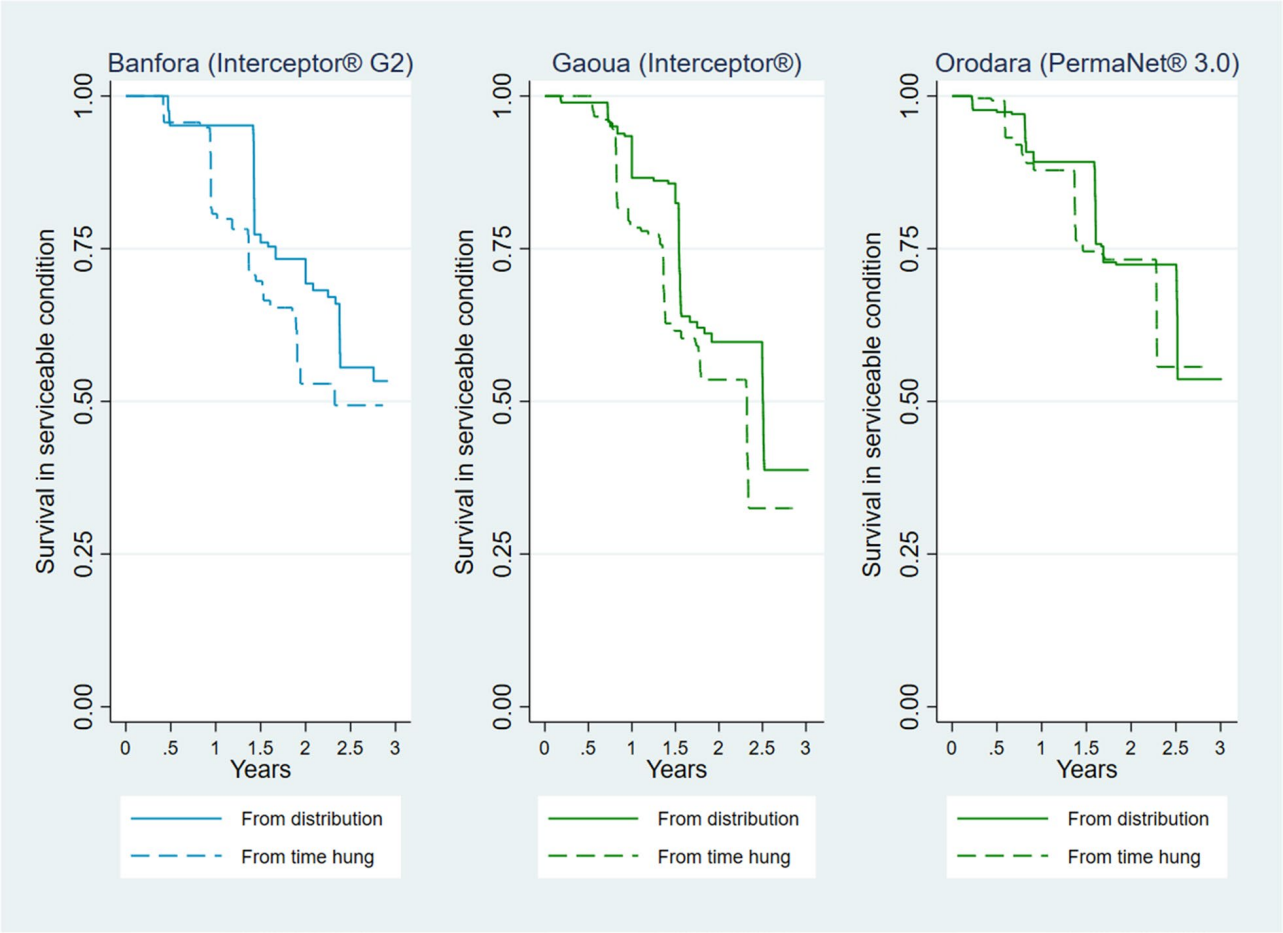
Kaplan–Meier curves for ITNs Surviving in Serviceable Condition in the Burkina Faso 2019–2022 Durability Monitoring of Interceptor^®^ G2, Interceptor^®^, and PermaNet^®^ 3.0

Kaplan–Meier survival curves were generated from the longitudinal data, and the curves considered failures (ITNs no longer in a serviceable condition due to attrition due to wear and tear, or physical survival) and censoring. Net survival was highest in Orodara and lowest in Gaoua when considering both time since distribution and time since first hanging (Fig. 5). After 3 years, the proportion of nets surviving was below 50% for Banfora and Gaoua, but the proportion remained above 50% for Orodara when considering risk as beginning at time the net was first hung. This was consistent with the median useful life estimates of less than three years in Gaoua and Banfora, but greater than three years for Orodara.

### Determinants of survival

The Cox Proportional household-level hazard model for determinants of Physical Durability is presented in Table S6. The study site, presence of children under the age of five, sex of the household head, SBC exposure, and observation of rodents were included in the final Cox regression analysis. Nets belonging to households who reported seeing rodents one or fewer times had a 38% lower risk of failure than nets belonging to households that reported always seeing rodents (aHR = 0.62, p < 0.001). Nets in households that received SBC messages at least once had a 31% lower risk of failure than those in households that had never received an SBC message (aHR = 0.69, p = 0.022). Nets belonging to households exposed twice or more to SBC messages had a 39% lower failure risk than nets belonging to households that were never exposed to SBC messages (aHR = 0.61, p = 0.003).

Nets in Orodara district were 42% less likely to fail than those in Banfora district (aHR = 0.58, p = 0.026). There was no significant association in the likelihood of failure between Gaoua district and Banfora district (aHR = 1.35, p = 0.156). Nets belonging to households who reported seeing rodents one or fewer times had a 34% lower failure rate than the nets belonging to households that reported seeing rodents more than once (aHR = 0.66, p = 0.005). Nets in households headed by females had a 34% lower risk of failure compared to households led by males (aHR = 0.66, p = 0.044). Nets in households exposed twice or more to SBC messages had a 48% reduction in the risk of failure compared to nets in households never exposed to SBC messages (aHR = 0.52,  ≤ 0.001). Similarly, nets belonging to households exposed once to SBC messages had a 36% lower risk failure than those never exposed (aHR = 0.64, p = 0.018). Nets that were ever folded up when not in use had a 53% lower risk of failure compared to those that were never folded up (aHR = 0.47, p < 0.001). Nets in households that reported a positive attitude towards net care once had a 32% lower risk of failure compared to those that never reported a positive attitude towards net care (aHR = 0.68, p = 0.024). Unexpectedly, the association was not significant for households where positive attitudes were recorded twice or more.

### Insecticidal efficacy

Mosquito strains were characterized prior to bioassay analysis (Table S7). The Kisumu strain was susceptible to both deltamethrin and alpha-cypermethrin, and polymerase chain reaction (PCR) confirmed the species to be *An. gambiae* with no mutations present. Resistance to both deltamethrin and alpha-cypermethrin was present in the VKPER strain. PBO synergist plus deltamethrin demonstrated 60% greater mortality than deltamethrin alone. Chlorenapyr characterization commence during the 12-month survey round. Across 12-, 24-, and 36-month survey round characterizations, 72-h mortality stayed above 95% with an interim diagnostic dose of 100 µg/bottle and 100% with 200 µg/bottle. PCR confirmed that the strain was *An. coluzzii* and had a high, but not fixed, frequency of knockdown resistance (KDR) west (L1014F) mutation but no presence of the *kdr east* (L1014S) mutation.

**Fig. 6:**
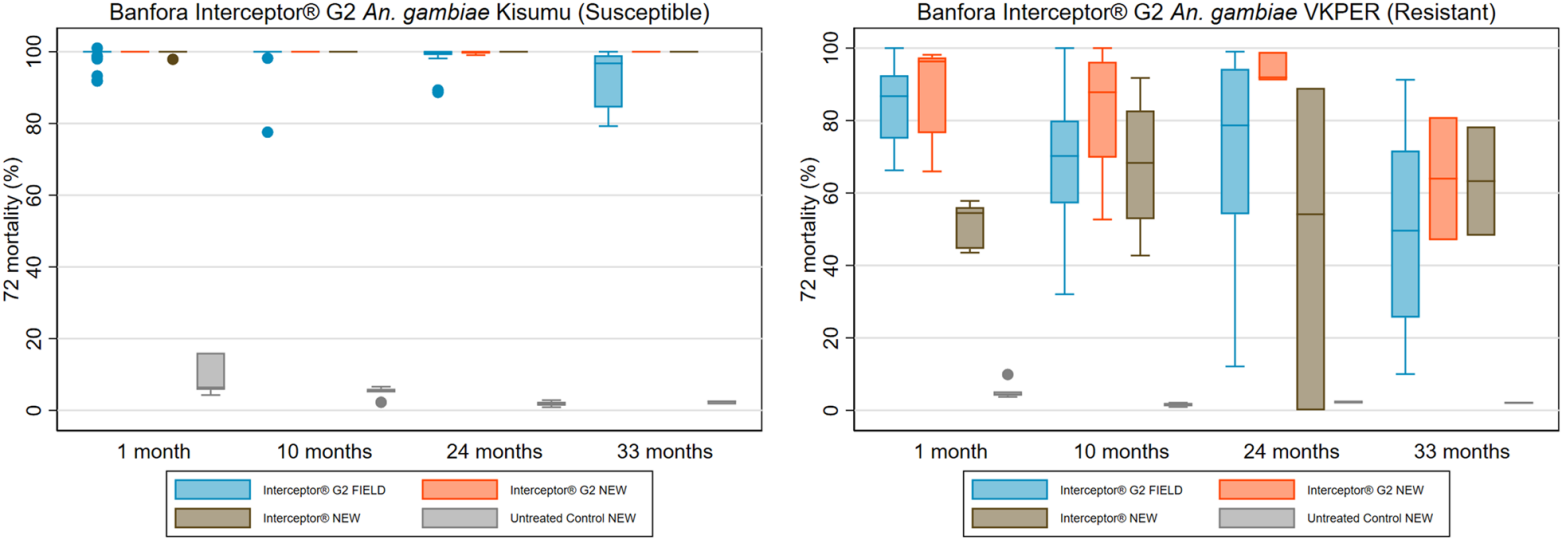
Box Plot of Interceptor^®^ G2 Tunnel Test Bioassay Results of Residual Efficacy of Alpha-Cypermethrin and Chlorfenapyr in Burkina Faso 2019–2022 ITNs Durability Monitoring

**Fig. 7:**
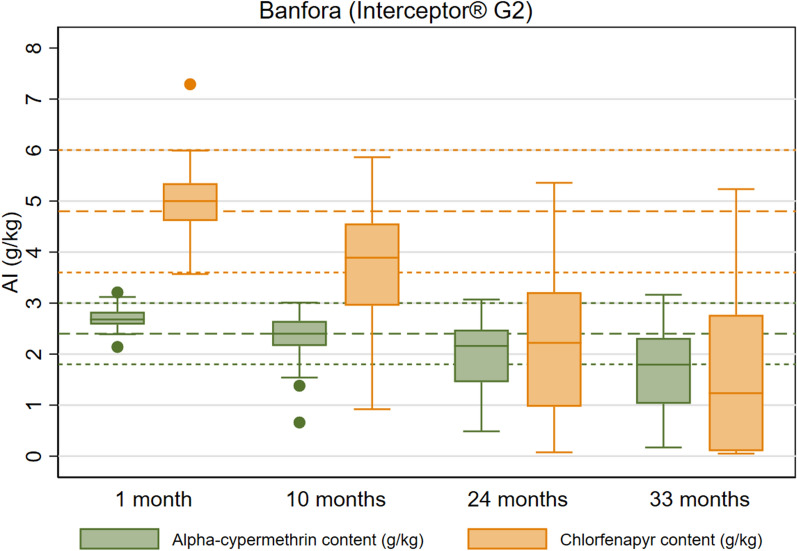
Box Plot of Interceptor^®^ G2 Chemical Content Results of Alpha-Cypermethrin and Chlorfenapyr in the Burkina Faso 2019–2022 ITN Durability Monitoring Study

Interceptor^®^ G2 arm: Tunnel tests against pyrethroid-susceptible mosquitoes *An. gambiae* Kisumu among the Interceptor^®^ G2 field samples indicated consistently high 72-h mortality across rounds (99% in the first three rounds and 93% (95% CI 90.4–96.1) at 36 months (Fig. 6 and Table S8). Against the pyrethroid-resistant *An. coluzzii* VKPER strain, field samples had a mean 72-h mortality of 51% (95% CI 39.5–61.4) at study endline, down from 85% (95% CI 79.7–89.3) at baseline. At study endline, mean alpha-cypermethrin content was 1.7 g/kg (95% CI 1.4–2.0), a reduction of 29% compared to the manufacturer’s target dose (2.4 g/kg) (Fig. 7 and Table S8). Mean chlorfenapyr content was 1.6 g/kg (95% CI 1.1–2.1), a 67% reduction compared to the 4.8 g/kg of the manufacturer target dose.

**Fig. 8:**
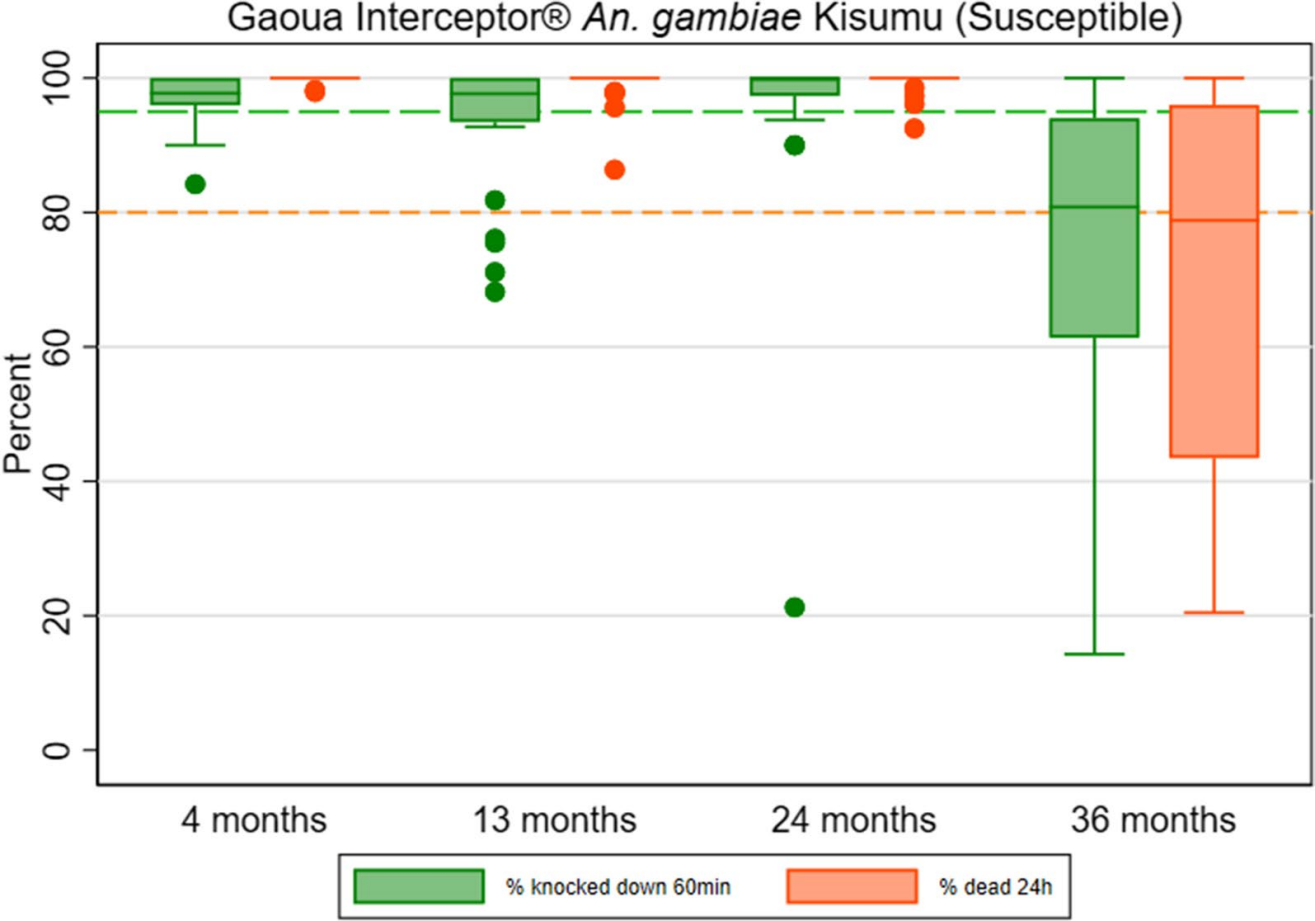
Box Plot of Interceptor^®^ Cone Bioassay Results for Residual Efficacy of Pyrethroid in the Burkina Faso 2019–2022 ITN Durability Monitoring Study

**Fig. 9:**
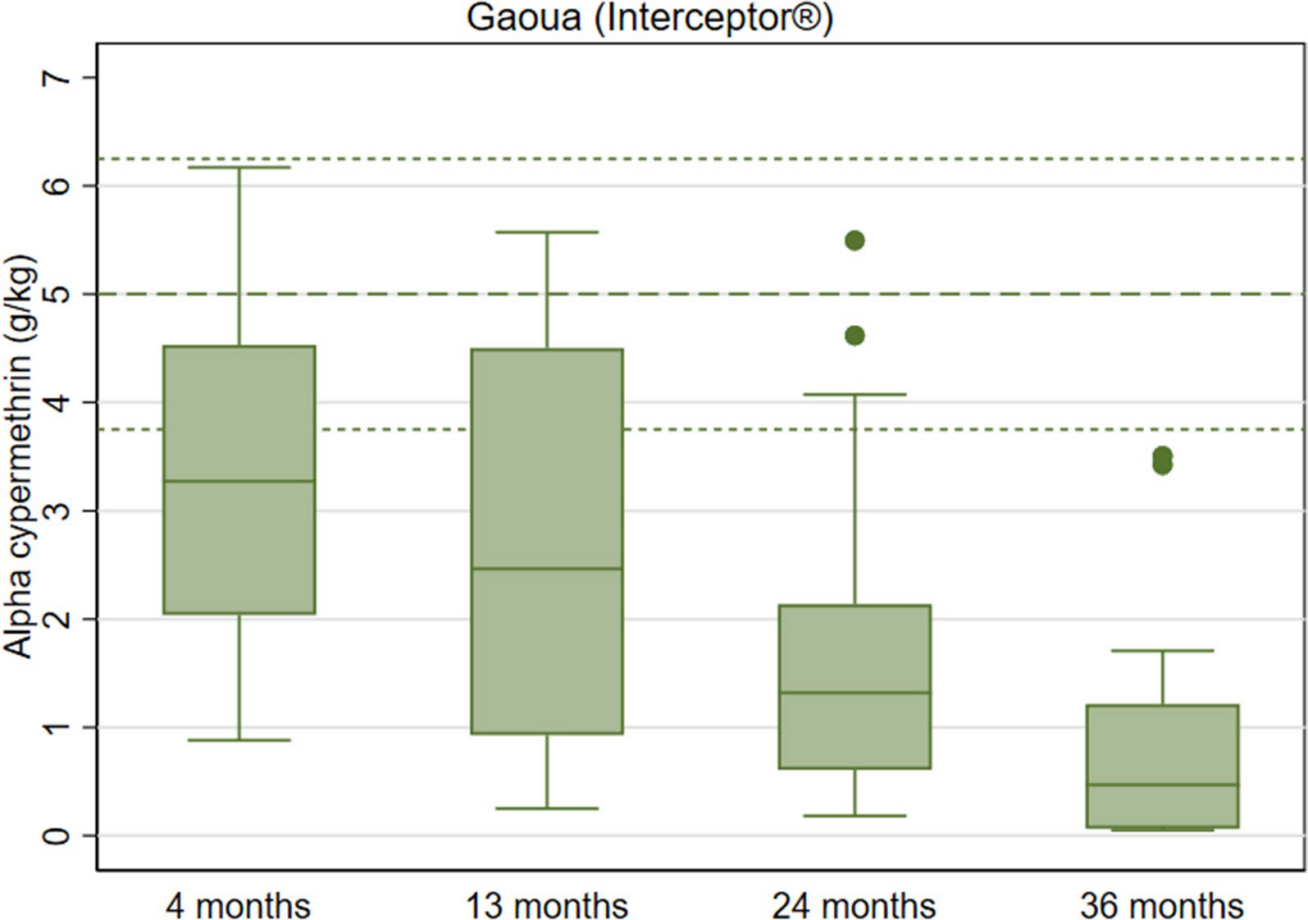
Box Plot of Interceptor^®^ Chemical Content Results of Pyrethroid in the Burkina Faso 2019–2022 ITN Durability Monitoring Study

Interceptor^®^ arm: during the first three rounds of data collection, the mean 24-h mortality obtained with *An*. *gambiae* Kisumu, the laboratory susceptible strain, was higher than 99% but declined to 71% (95% CI 58.0–83.7) at study endline (Fig. 8 and Table S8). The proportion of nets that were optimally effective declined from 100% in third round to 60% (95% CI 37.2–82.8) by study endline. The proportion of nets with minimal effectiveness decreased from 100 to 87% (95% CI 70.4–100.0 by study endline. After 36 months of use in the field, the mean alpha-cypermethrin content was 0.8 g/kg (95% CI 0.3–1.3), an 84% reduction compared to the manufacturer threshold for alpha-cypermethrin of 5.0 g/kg, (Fig. 9 and Table S8).

**Fig. 10:**
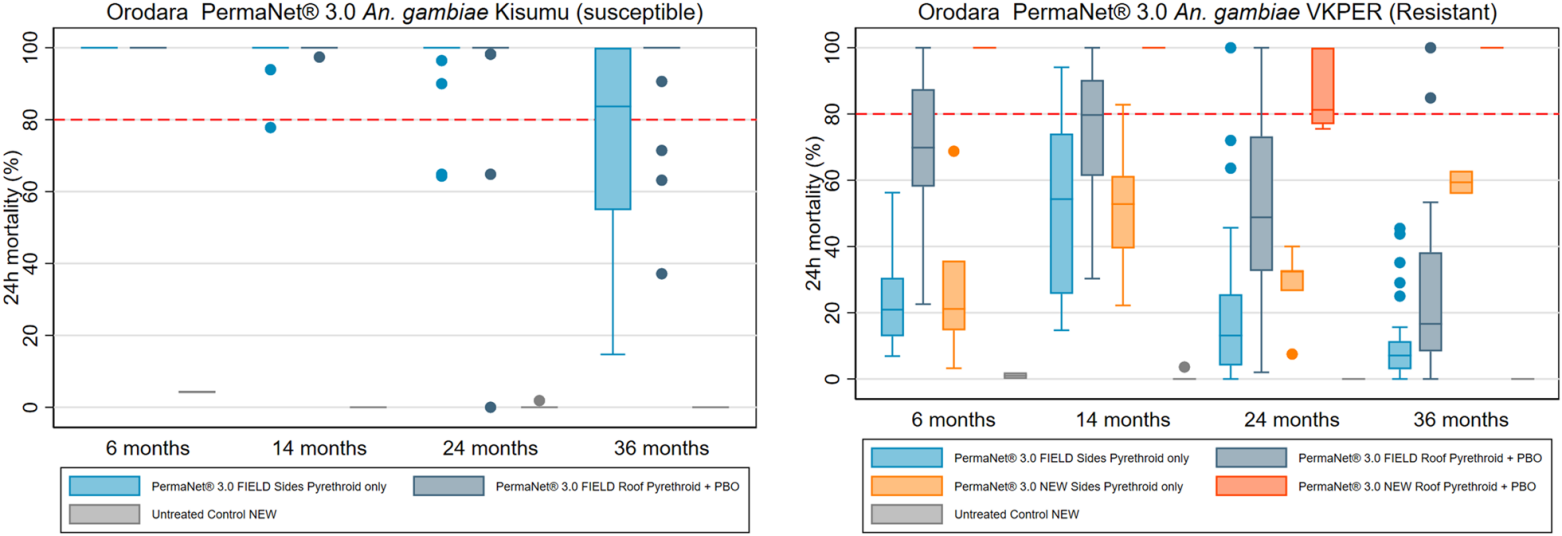
Box Plot of PermaNet^®^ 3.0 Cone Bioassay Results for Residual Efficacy of Pyrethroid and PBO in the Burkina Faso 2019–2022 ITN Durability Monitoring Study

**Fig. 11:**
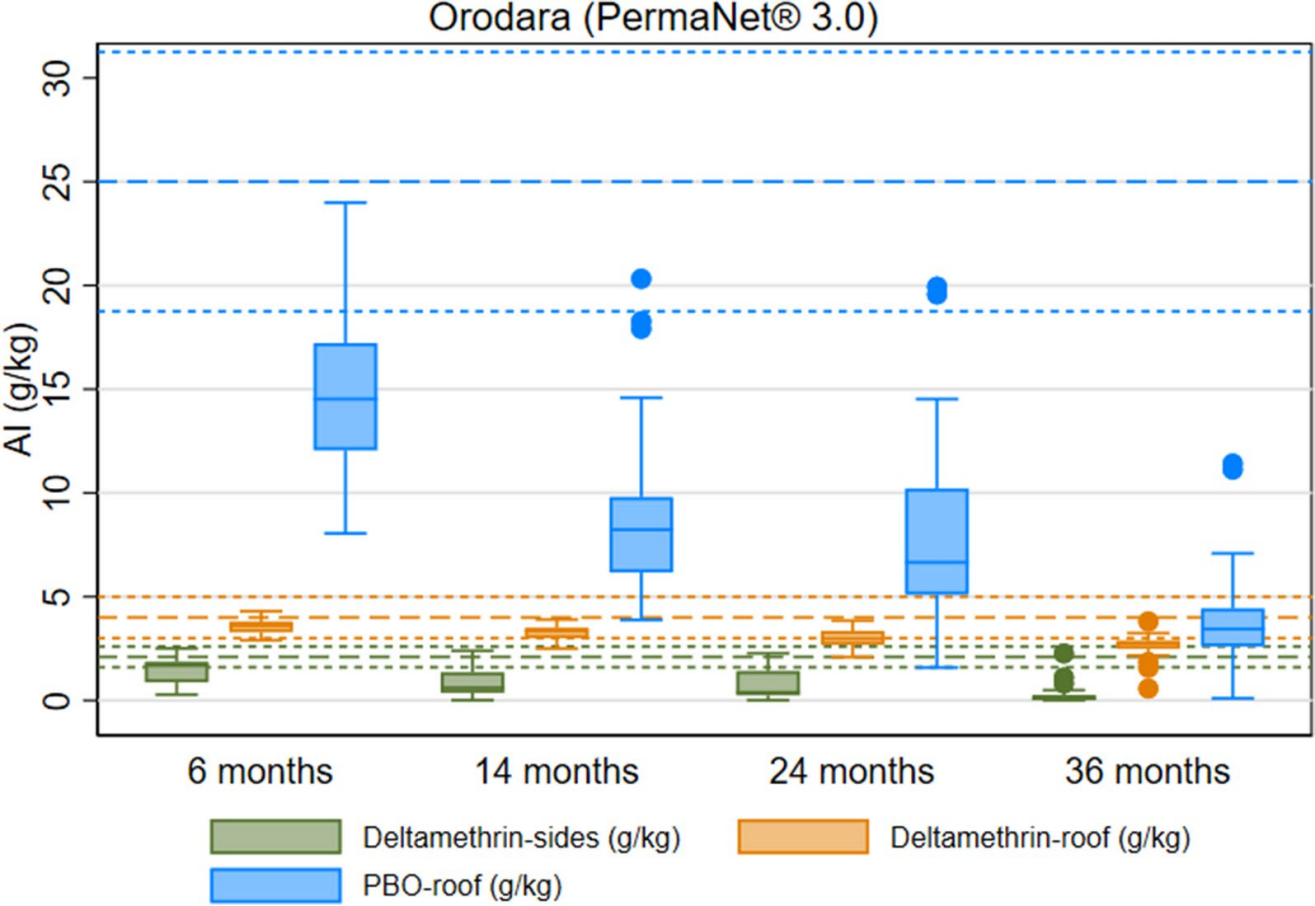
Box Plot of PermaNet^®^ 3.0 Chemical Content Results of Pyrethroid and PBO in the Burkina Faso 2019–2022

PermaNet^®^ 3.0 arm: Samples of the side panels (containing deltamethrin-only) showed 72% (95% CI 61.3–82.5) 24-h mortality against pyrethroid-susceptible *An. gambiae* Kisumu mosquitoes 36 months after distribution. Mortality in roof panel samples (which incorporated a PBO-synergist), achieved 95% (95% CI 90.2–100.0) 24-h mortality (Fig. 10 and Table S8). Against resistant mosquitoes from *An. coluzzii* VKPER strain, roof samples had higher mean 24-h mortality outcomes than pyrethroid-only side samples (26% (95% CI 15.8–35.5) vs. 11% (95% CI 6.6–15.1)). After 36 months of use in the field, the mean deltamethrin content of side panels was 0.3 g/kg (95% CI 0.1–0.4), an 86% loss compared to the 2.1 g/kg manufacturer target dose (Fig. 11 and Table S8). The mean deltamethrin content on roof samples was 2.6 g/kg (95% CI 2.4–2.9), a 35% loss compared to 4.0 g/kg of manufacturer target dose. At study endline, the chemical content of PBO on roof samples was 3.9 g/kg (95% CI 3.0–4.8), an 84% loss compared to the manufacturer's target dose of 25.0 g/kg.

## Discussion

This study evaluated the physical and insecticidal integrity of three ITN brands: PermaNet^®^ 3.0 in Orodara, standard Interceptor^®^ in Gaoua and Interceptor^®^ G2 in Banfora, following a 2019 mass campaign distribution in Burkina Faso. At study endline, 54% (95% CI 41.4–65.8) of cohort ITNs in Orodara, 48% (95% CI 35.6–59.7) in Banfora, and 37% (95% CI 27.9–47.4) in Gaoua survived and were in serviceable condition, corresponding to 3.2 (95% CI 2.5–4.0) years of median survival for PermaNet^®^ 3.0 in Orodara, 2.6 (95% CI 1.9–3.2) years for the Interceptor^®^ G2 in Banfora, and 2.4 (95% CI 1.9–2.9) years for Interceptor^®^ in Gaoua. The differences in ITN brand survival between districts was not statistically significant (p = 0.47). Pyrethroid-only Interceptor^®^ ITNs in Gaoua and dual AI ITNs Interceptor^®^ G2 in Banfora did not achieve a three-year median life, which is the WHO’s universal recommendation [3]. The lower survival rate and median useful life of Interceptor^®^ ITNs in Gaoua can be explained by higher attrition (41%) due to wear and tear compared to 31% for PermaNet^®^ 3.0 ITNs in Orodara, and 20% for Interceptor^®^ G2 ITNs in Banfora (p = 0.013). Additionally, Interceptor^®^ ITNs in Gaoua also had the highest joint percentage of torn nets (20%) along with Interceptor^®^ G2 ITNs in Banfora, compared to PermaNet 3.0 ITNs in Orodara (10%). In Gaoua, the leading cause of attrition contributing to wear and tear was the throwing away of a net when it was too torn (38%), alternative use of the net (3%), and the ITN being destroyed (< 1%). In Orodara, ITNs that were destroyed (14%) and thrown away (13%) were the primary wear and tear-related causes of attrition. In Banfora, nets lost to wear, and tear comprised 16% of ITNs that were thrown away and 4% that were destroyed. Recent publications on durability monitoring evaluating pyrethroid-only ITNs (DawaPlus^®^ 2.0 in three States in Nigeria, Olyset^®^ and PermaNet^®^ 2.0 in Zanzibar, and MAGNet^®^ and Royal Sentry^®^ in three ecological zones in Mozambique) showed attrition rates due to wear and tear varying by district from 8 to 24%, corresponding to median useful life estimates of greater than three years [7, 11, 12]. Generally, the lower the attrition rate due to wear and tear, the greater the median useful life (> 3 years), a trend that was also seen in this study. At study endline, the proportion of ITNs classified as still serviceable with regards to physical integrity was consistently high across the three study sites (80% in Gaoua and Banfora, compared to 90% in Orodara). In Orodara and Banfora, the combination of a higher serviceability and a lower attrition due to wear and tear resulted in a higher survival rate and estimated median useful life.

A total of 923 labelled ITNs were enrolled at baseline (347 in Orodara, 294 in Banfora, and 282 in Gaoua). Banfora had the highest all-cause attrition rate (70%), followed by Gaoua (64%) and Orodara (55%). Nets that underwent attrition for unknown reasons, including nets given away to relatives, nets given away to others, nets stolen, sold, or lost for unknown reasons were excluded from the survival analysis. In total, 193 ITNs (66%) in Banfora, 80 ITNs (28%) in Gaoua and 111 ITNs (32%) in Orodara were excluded. Several variables were included in the final household and net-level models used for the cox regression analysis to explain the survival determinants of the three ITN brands during their three years of field use. At the cohort net-level, study site was identified as a significant determinant, revealing that nets in Orodara lasted longer than nets in Banfora. Given that the study sites are similar with respect to environmental, epidemiological, and population profiles, the significant differences between sites were likely related to the behavior of the population.

Rodents are often attracted to food stored in or around a household. When food is stored in a room used for sleeping, the risk of damage to ITNs caused by rodents increases. For both household-level and net-level models, never seeing rodents was identified as a determinant for net survival. While rodents have been shown to be a mechanism for net damage, as they are attracted to food being stored in rooms with nets [6, 8, 12], the practice of always storing food in the room used for sleeping was not a significant determinant in this study’s cox survival analysis.

Nets belonging to houses headed by females were more protective to ITN durability than nets in households headed by males, similar to finding elsewhere [39]. In both the household-level and net-level models, SBC exposure was a determinant of increased net survival, a finding that is well established in the literature [5, 7, 10–12]. In the net-level model, repeated exposure to SBC messages (twice or more) decreased the risk of net failure by 48%, while a single exposure reduced the risk by 36%. Positive attitude toward net care was highly correlated to SBC exposure (coefficient = 0.2854, p < 0.001), indicating that regular and sustained SBC exposure increases positive net care attitudes. In the net-level model, households with a positive attitude toward net care was a predictor of increased net durability. Conversely, in Gaoua, where a low proportion of respondents had favorable attitudes towards net care (18%), it is possible that a lack of SBC messaging on the benefits of net use, care, and repair, contributed to lower attitude scores, and in turn, to lower ITN survival.

Folding nets while not in use is a common determinant of reduced risk of net risk failure [5, 7, 12]. The Cox analysis employed for this study identified that folding nets while not in use was protective, reducing the risk of net failure by 53%. The practice of always folding nets while not in use was highest in Gaoua (48%), followed by Banfora (28%) and Orodara (1%, p < 0.001).

This study presented a first opportunity to gain insight into the chemical performance of dual AI Interceptor^®^ G2 and PermaNet^®^ 3.0 ITNs after three years of field use. Mortality for both dual AI ITN brands against resistant mosquitoes was high in the initial survey rounds but decreased over time as chlorfenapyr and PBO content waned.

Mortality of pyrethroid-resistant *An. gambiae* in PermaNet^®^ 3.0 field samples was three times lower at 36 months than at baseline (26% vs. 72%). This decrease in mortality corresponds with an 84% loss in mean PBO content and 35% loss in mean deltamethrin content on roof samples, compared to the manufacturer’s target dose. After 24 months of field use, 24-h mortality on roof samples of PermaNet^®^ 3.0 ITNs against the resistant strain was 52%, lower than the 99% and 95% 24-h mortality observed at 24-months against two resistant strains during a recent durability monitoring study in Burundi (unpublished) [40].

After 36 months, Interceptor^®^ G2 field samples achieved 51% 72-h mortality against the resistant *An. coluzzii* VKPER strain. This decrease corresponds roughly to the 67% reduction in chlorfenapyr content and 29% loss of alpha-cypermethrin content compared to their original manufacturer’s target doses. The mean 24-h mortality of Interceptor^®^ ITNs recorded at endline (71%) dropped below the WHO threshold of 80% [36]. This was likely due to the 84% reduction in the chemical concentration of alpha-cypermethrin in sampled nets compared to the original target dose (5.0 g/kg). Optimal effectiveness of Interceptor^®^ field samples decreased from 100% at baseline to 60% at endline round. Several factors have been observed on insecticidal efficacy of standard, pyrethroid-only ITNs after three years of field use. Depending on net brand, optimal effectiveness ranging from 29 to 97% have been reported in different countries [7, 11, 12].

This ITN durability monitoring study was not a national-level representation of ITN retention but provide some evidence that suggest that ITN retention rate in Burkina Faso may exceed three years for Banfora and Orodara, where dual AI (Interceptor® G2 and PermaNet^®^ 3.0) were distributed.

## Limitations

In an effort to assure appropriate precautions were taken to prevent the transmission of COVID-19, beginning in the second survey round, interviewers did not enter study houses to observe the household’s net hanging behavior or practices related to storing or cooking food in the room used for sleeping. Instead, interviewers relied upon the declaration of the household respondents, which could not be confirmed. Data was not always collected during the same season across districts. For example, during the 24-month survey round, data was collected during the rainy season in Orodara and Gaoua, and the dry season in Banfora. This may have affected ITN durability risk factors and use data. ITN brands were assigned to separate districts so, the study could not be designed to examine multiple ITN brands in a single district. This could have potentially introduced confounding related to inter-district variability. To minimize bias, the three study districts were selected from the same region and had similar epidemiological, climatic and population profiles. Additionally, the Hawthorne effect has the potential to introduce bias in research outcomes if the act of tagging and labeling nets within a household induces modifications in the behaviors of household members towards these nets. Finally, apart from the endline round, nets withdrawn for bioassay and chemical analysis were not cohort nets, but rather were collected from the neighboring households surrounding the cohort household. Therefore, the duration of net use for these nets was unknown and could influence the insecticidal efficacy results.

## Conclusion

Based on the median useful ITN life after 36 months of field use, dual AI PermaNet^®^ 3.0 ITNs achieved higher median useful life than the estimates standard of three years while dual AI Interceptor^®^ G2 and pyrethroid-only Interceptor^®^ ITNs did not.

While the observation of rodents in sleeping areas was identified as a contributing factor for decreased ITN survival, storage of food in sleeping areas, which attracts rodents, was not. In future durability monitoring studies, variables related to factors attracting rodents, beyond the storage of food in the room used for sleeping, could be investigated. Receipt of SBC messaging as well as women-headed households were shown to be protective for ITN durability.

Declines in the insecticidal effectiveness of PermaNet^®^ 3.0 and Interceptor^®^ G2 ITNs against resistant mosquito strains were driven by losses of PBO and chlorfenapyr, respectively. Pyrethroid-only Interceptor^®^ ITNs performed consistently high against a pyrethroid-susceptible strain during the first three survey rounds (optimal effectiveness 100%) but declined to 60% at study endline round, likely due to a reduction of the alpha-cypermethrin concentration.

Durability monitoring conducted in Burkina Faso revealed that dual AI Interceptor^®^ G2 lasted for 2.6 years while PermaNet^®^ 3.0 ITNs was sufficiently durable, exceeding the three-year survival expectation under field conditions. This study’s insecticidal efficacy results for Interceptor® G2 and PermaNet^®^ 3.0 brands demonstrated that initially high levels of mortality decreased over time due to a loss of chlorfenapyr and PBO. Additional data on field performance of dual AI nets from other countries can complement the findings from Burkina Faso.

## Disclaimer

The findings and conclusions expressed herein are those of the authors and do not necessarily represent the official position of USAID, PMI, nor the U.S. Centers for Disease Control and Prevention (CDC).

### Supplementary Information

Additional file 1.

## Data Availability

All data generated or analyzed during this study are included in this published article as additional files.

## Abbreviations

**aHR**: Adjusted hazard ratio
**AI**: Active ingredient
**BFI**: Blood feeding inhibition
**CI**: Confidence interval
**HR**: Hazard ratio
**IQR**: Interquartile range
**IRSS**: Institut de Recherche en Sciences de la Santé
**ITN**: Insecticide-treated net
**KDR**: Knockdown resistance
**KD60**: 60-minute knock-down
**NMP**: National Malaria Programme
**NNP**: New Nets Project
**ODK**: Open Data Kit
**PBO**: Piperonyl butoxide
**pHI**: Proportionate Hole Index
**PMI**: President’s Malaria Initiative
**SBC**: Social and behavior change
**SP-Palu**: Permanent Secretariat for Malaria Elimination
**WHO**: World Health Organization
