# Supplementary material for: Physical and insecticidal durability of Interceptor®, Interceptor® G2, and PermaNet® 3.0 insecticide-treated nets in Burkina Faso: results of durability monitoring in three sites from 2019 to 2022
Source: Malar J. 2024 Jun 4;23:173. doi: 10.1186/s12936-024-04989-w (PMC11149234; doi:10.1186/s12936-024-04989-w)
Supplement: Supplementary file 1 — Additional file 1. [file 12936_2024_4989_MOESM1_ESM.docx]

# Additional Files

*Table 1. Follow-up status of households and cohort ITNs at study endline in Banfora, Gaoua and Orodora*

| **Variable** | **Banfora**  **(Interceptor® G2) % (95%CI)** | | **Gaoua**  **(Interceptor®) % (95% CI)** | | **Orodara**  **(PermaNet® 3.0) % (95% CI)** | |
| --- | --- | --- | --- | --- | --- | --- |
| **Households** | **N=170** | | **N=170** | | **N=170** | |
| Still has any cohort ITN | 29.4 | (19.5-41.7) | 45.3 | (35.5-55.4) | 60.6 | (49.4-70.8) |
| Lost all their cohort ITNs | 31.8 | (21.3-44.4) | 40.6 | (32.7-48.9) | 19.4 | (13.8-26.5) |
| Moved away | 20.6 | (13.9-29.5) | 8.2 | (4.1-15.7) | 17.0 | (11.9-23.8) |
| Refused | 1.2 | (0.3-4.6) | 1.2 | (0.3-4.6) | 0.6 | (0.1-4.3) |
| Nobody home at survey or not reached | 0.6 | (0.1-4.3) | 4.7 | (1.9-11.3) | 2.4 | (0.9-5.7) |
| Unknown status | 16.4 | (5.3-40.8) | 0.0 | (--) | 0.0 | (--) |
| **Labelled cohort ITNs** | **N=294** | | **N=282** | | **N=347** | |
| Known outcome | 34.4 | (24.1-46.3) | 71.6 | (64.3-78.0) | 68.0 | (49.4-70.8) |
| Unknown outcome | 65.6 | (53.7-75.9) | 28.4 | (22.0-35.7) | 32.0 | (13.8-26.5) |
| Household moved away or refused | 19.0 | (14.2-25.0) | 6.7 | (3.2-13.5) | 13.5 | (11.9-23.8) |
| Net given away or used elsewhere | 28.6 | (19.3-40.0) | 12.8 | (9.2-17.5) | 10.4 | (0.1-4.3) |
| Fate of net unknown | 18.0 | (7.0-39.1) | 8.9 | (5.4-14.3) | 8.1 | (5.7-11.3) |
| 27 households in Banfora District could not be visited at the third round and study endline because of insecurity in three study clusters. | | | | | | |

*Table 2. Baseline Characteristics of Households Enrolled in the Burkina Faso 2019-2022 ITNs Durability Monitoring of Interceptor® G2, Interceptor®, and PermaNet® 3.0*

| **Variable** | **Banfora (Interceptor® G2) % (95%CI)** | | **Gaoua (Interceptor®) % (95% CI)** | | **Orodara (PermaNet® 3.0) % (95% CI)** | | **p-value** |
| --- | --- | --- | --- | --- | --- | --- | --- |
| **Demographic** |  |  |  |  |  |  |  |
| Mean number of household members | 3.4 | (3.1-3.8) | 4.3 | (3.9-4.7) | 4.5 | (4.1-4.9) | <0.001 |
| Mean age of head of household (years) | 43.8 | (40.5-47.1) | 46.5 | (43.2-49.8) | 41.7 | (39.2-44.2) | 0.297 |
| Percentage of female headed households | 18.2 | (12.5-25.8) | 28.7 | (20.9-38.1) | 12.4 | (7.9-18.7) | 0.004 |
| Education of head of household (male) |  |  |  |  |  |  |  |
| Non-literature | 54.1 | (40.4-67.2) | 75.4 | (58.4-87.1) | 74.5 | (63.8-82.9) | 0.012 |
| Primary | 28.9 | (19.4-40.7) | 7.9 | (3.6-16.5) | 18.1 | (11.9-26.6) |  |
| Secondary | 17.0 | (9.1-29.5) | 16.7 | (8.1-31.1) | 7.4 | (3.8-13.9) |  |
| Education of head of household (female) |  |  |  |  |  |  |  |
| Non-literature | 83.3 | (61.5-94.0) | 89.1 | (72.9-96.2) | 90.5 | (68.6-97.6) | 0.449 |
| Primary | 6.7 | (1.6-24.3) | 8.7 | (2.4-26.6) | 0.0 | (--) |  |
| Secondary | 10.0 | (3.4-25.9) | 2.2 | (0.3-13.7) | 9.5 | (2.4-31.4) |  |
| **Housing characteristics** |  |  |  |  |  |  |  |
| Improved roof materials | 91.8 | (82.1-96.4) | 79.4 | (68.9-87.0) | 95.9 | (90.6-98.2) | 0.002 |
| Improved wall materials | 44.7 | (28.7-61.9) | 37.1 | (22.0-55.2) | 29.4 | (18.4-43.5) | 0.393 |
| Improved floor materials | 81.2 | (68.5-89.5) | 55.9 | (41.8-69.1) | 64.1 | (53.1-73.8) | 0.013 |
| Cook with charcoal (vs firewood) | 13.7 | (4.4-35.2) | 6.7 | (1.3-27.4) | 1.8 | (0.6-5.1) | 0.157 |
| Access to safe water source | 90.6 | (68.1-97.7) | 90.6 | (68.1-97.7) | 98.8 | (95.4-99.7) | 0.271 |
| Access to any latrine | 75.3 | (56.2-87.9) | 31.8 | (18.2-49.4) | 93.5 | (87.2-96.8) | <0.001 |
| **Household assets** |  |  |  |  |  |  |  |
| Any transport | 84.7 | (78.4-89.4) | 65.9 | (56.1-74.5) | 95.3 | (91.1-97.6) | <0.001 |
| Type of transport |  |  |  |  |  |  |  |
| Bicycle | 70.0 | (59.1-79.0) | 52.9 | (43.1-62.6) | 84.7 | (76.4-90.4) | <0.001 |
| Moto | 61.8 | (53.6-69.3) | 41.2 | (31.1-52.0) | 77.1 | (69.0-83.5) | <0.001 |
| Car | 0.6 | (0.1-4.3) | 2.4 | (0.5-10.6) | 2.9 | (1.1-7.6) | 0.407 |
| Owns livestock | 62.9 | (47.1-76.4) | 73.5 | (57.9-84.9) | 59.4 | (46.5-71.2) | 0.334 |
| Type of livestock |  |  |  |  |  |  |  |
| Chicken | 54.1 | (39.3-68.3) | 65.3 | (50.7-77.5) | 55.9 | (43.5-67.6) | 0.465 |
| Goat | 50.0 | (34.2-65.8) | 61.8 | (47.0-74.6) | 42.9 | (32.1-54.5) | 0.173 |
| Pig | 5.9 | (2.0-16.1) | 34.7 | (23.8-47.5) | 0.6 | (0.1-4.3) | <0.001 |
| Items owned by household |  |  |  |  |  |  |  |
| Radio | 70.0 | (61.4-77.4) | 38.8 | (26.5-52.8) | 76.5 | (67.0-83.9) | <0.001 |
| TV | 27.6 | (15.4-44.4) | 15.3 | (6.1-33.2) | 32.9 | (22.2-45.9) | 0.196 |
| Simple mobile | 84.1 | (75.7-90.0) | 64.1 | (51.7-74.9) | 92.9 | (84.2-97.0) | <0.001 |
| Smartphone | 8.2 | (3.7-17.5) | 8.8 | (2.9-24.2) | 14.1 | (6.5-27.9) | 0.581 |
| p-value for overall comparison of difference between sites; test for trend for education comparisons.  (--): No 95% CI | | | | | | | |

*Table 3. Household Level Risk Factors Across Survey Rounds*

|  | **Banfora**  **(Interceptor® G2) % (95%CI)** | | **Gaoua (Interceptor®) % (95% CI)** | | **Orodara**  **(PermaNet® 3.0) % (95% CI)** | | **P-value** |
| --- | --- | --- | --- | --- | --- | --- | --- |
|  | **N=170** | | **N=170** | | **N=170** | |  |
| **Observed rodents in past six months** | | | | | | | |
| Never | 32.4 | (16.3-54.0) | 1.2 | (0.3-4.6) | 4.1 | (2.0-8.4) | <0.001 |
| At times | 32.9 | (21.6-46.7) | 42.9 | (28.0-59.3) | 48.2 | (35.6-61.1) |  |
| Always | 34.7 | (20.3-52.6) | 55.9 | (39.2-71.3) | 47.6 | (35.4-60.1) |  |
| **Storing food in sleeping rooms** | | | | | | | |
| Never | 37.1 | (24.3-52.0) | 4.7 | (2.0-10.5) | 26.5 | (17.4-38.1) | <0.001 |
| At times | 48.2 | (36.4-60.3) | 39.4 | (28.3-51.7) | 60.0 | (51.5-67.9) |  |
| Always | 14.7 | (7.3-27.6) | 55.9 | (42.8-68.2) | 13.5 | (7.0-24.6) |  |
| **Cooking in sleeping rooms** | | | | | | | |
| Never | 80.6 | (67.7-89.2) | 37.6 | (26.9-49.8) | 81.8 | (74.3-87.4) | <0.001 |
| At times | 19.4 | (10.8-32.3) | 61.8 | (49.7-72.5) | 18.2 | (12.6-25.7) |  |
| Always | 0.0 | (--) | 0.6 | (0.1-4.3) | 0.0 | (--) |  |
| **Exposure to net use or care messages** | | | | | | | |
| Never | 34.7 | (19.8-53.4) | 58.2 | (37.7-76.3) | 26.5 | (15.1-42.1) | 0.001 |
| Once | 31.8 | (21.5-44.1) | 34.1 | (19.9-51.8) | 21.2 | (12.9-32.7) |  |
| Twice or more | 33.5 | (20.6-49.5) | 7.6 | (3.2-17.4) | 52.4 | (32.4-71.6) |  |
| **Very positive net use attitude (score > 1.0)** | | | | | | | |
| Never | 20.6 | (12.8-31.5) | 66.5 | (54.4-76.7) | 8.2 | (3.6-17.9) | <0.001 |
| Once | 37.1 | (28.2-46.8) | 27.6 | (20.1-36.7) | 30.0 | (21.6-40.0) |  |
| Twice or more | 42.4 | (29.8-56.0) | 5.9 | (3.0-11.2) | 61.8 | (47.9-73.9) |  |
| **Very positive net care attitude (score > 1.0)** | | | | | | | |
| Never | 54.1 | (34.4-72.6) | 82.4 | (76.1-87.3) | 35.3 | (26.2-45.6) | <0.001 |
| Once | 19.4 | (12.8-28.4) | 15.3 | (11.0-20.9) | 44.1 | (36.6-51.9) |  |
| Twice or more | 26.5 | (14.6-43.0) | 2.4 | (0.9-5.7) | 20.6 | (13.9-29.5) |  |
| p-value from test for trend for overall comparison between sites | | | | | | | |

*Table 4. Net-Level Risk Factors Across All Survey Rounds*

|  | **Banfora (Interceptor® G2) % (95%CI)** | | **Gaoua (Interceptor®) % (95% CI)** | | **Orodara (PermaNet® 3.0) % (95% CI)** | | **P-value** |
| --- | --- | --- | --- | --- | --- | --- | --- |
| **All labelled cohort ITNs** | **N=294** | | **N=282** | | **N=347** | |  |
| Ever observed / reported hanging | 68.7 | (58.4-77.4) | 81.6 | (74.5-87.0) | 88.2 | (80.3-93.2) | 0.002 |
| Ever reported used | 73.5 | (62.4-82.2) | 84.0 | (78.3-88.5) | 89.3 | (80.1-94.6) | 0.016 |
| Ever reported washed | 50.7 | (37.5-63.7) | 78.0 | (71.1-83.7) | 81.0 | (72.6-87.3) | <0.001 |
| **Cohort ITN ever hung** | **N=202** | | **N=230** | | **N=306** | |  |
| **Tied up or folded when hanging** |  |  |  |  |  |  |  |
| Never | 50.5 | (31.8-69.1) | 21.7 | (12.4-35.3) | 93.8 | (90.4-96.0) | <0.001 |
| At times | 21.3 | (12.8-33.3) | 30.0 | (21.9-39.6) | 4.9 | (3.0-7.8) |  |
| Always | 28.2 | (16.5-43.9) | 48.3 | (31.6-65.3) | 1.3 | (0.4-4.8) |  |
| **Type of sleeping place** | **N=213** | | **N=239** | | **N=312** | |  |
| Reed mat / Ground | 46.9 | (37.6-56.5) | 83.3 | (62.6-93.7) | 27.9 | (18.5-39.8) | <0.001 |
| Bed frame (unfinished) | 15.0 | (8.6-24.9) | 0.8 | (0.2-3.2) | 35.6 | (26.3-46.1) |  |
| Bed frame (finished) | 17.8 | (11.4-26.8) | 5.0 | (1.5-15.9) | 20.5 | (14.4-28.3) |  |
| Foam mattress | 20.2 | (12.6-30.8) | 10.9 | (3.4-29.6) | 16.0 | (9.8-25.1) |  |
| **Cohort ITN ever used** | **N=216** | | **N=237** | | **N=310** | |  |
| **Net was used by** |  |  |  |  |  |  |  |
| Children only | 4.9 | (2.9-8.3) | 0.9 | (0.2-3.4) | 2.6 | (1.3-5.3) | 0.002 |
| Children with adults | 51.7 | (42.6-60.7) | 52.8 | (47.6-58.0) | 65.9 | (59.8-71.5) |  |
| Adults only | 43.3 | (33.2-54.1) | 46.3 | (40.9-51.9) | 31.5 | (26.3-37.2) |  |
| **Cohort ITN ever washed** | **N=149** | | **N=220** | | **N=281** | |  |
| Washes last 6 months (Median [IQR]) | 2.0 | [1.0-2.5] | 2.0 | [2.0-3.0] | 2.0 | [1.0-3.5] | <0.001 |
| **Use of detergent** |  |  |  |  |  |  |  |
| Never | 61.1 | (41.3-77.8) | 36.4 | (28.1-45.5) | 65.1 | (57.2-72.3) | <0.001 |
| At times | 16.1 | (10.5-23.9) | 37.3 | (29.9-45.3) | 29.9 | (22.5-38.4) |  |
| Always | 22.8 | (11.8-39.4) | 26.4 | (19.3-34.9) | 5.0 | (2.5-9.8) |  |
| **Drying net outside** |  |  |  |  |  |  |  |
| Never | 1.3 | (0.4-4.9) | 1.4 | (0.2-9.5) | 2.8 | (0.9-8.2) | 0.013 |
| At times | 1.3 | (0.3-5.1) | 7.3 | (3.9-13.1) | 16.4 | (8.3-29.6) |  |
| Always | 97.3 | (92.0-99.1) | 91.4 | (84.6-95.3) | 80.8 | (65.0-90.5) |  |
| **Drying over bush or fence** |  |  |  |  |  |  |  |
| Never | 91.9 | (80.2-97.0) | 68.2 | (53.1-80.2) | 86.1 | (75.6-92.5) | 0.004 |
| At times | 5.4 | (2.1-13.0) | 20.5 | (14.8-27.6) | 11.0 | (5.5-20.9) |  |
| Always | 2.7 | (0.8-9.1) | 11.4 | (5.2-23.0) | 2.8 | (1.3-5.9) |  |
| p-value for overall comparison between districts IQR: interquartile range | | | | | | |  |

*Table 5. Physical Integrity of Observed Cohort ITNs*

|  | **First round % (95% CI)** | | **Second round % (95% CI)** | | **Third round % (95% CI)** | | **Study endline % (95% CI)** | |
| --- | --- | --- | --- | --- | --- | --- | --- | --- |
| **Banfora (Interceptor® G2)** | **N=282** | | **N=231** | | **N=101** | | **N=60** | |
| Mean months since campaign | 1.4 | | 10.1 | | 24.1 | | 33.1 | |
| Net has any hole | 3.1 | (1.3-6.9) | 27.7 | (21.7-34.7) | 69.3 | (57.5-79.0) | 83.3 | (72.6-90.4) |
| **Physical condition (pHI)** |  |  |  |  |  |  |  |  |
| Good (0 - 64) | 100.0 | (--) | 91.8 | (87.1-94.9) | 68.3 | (57.4-77.6) | 51.7 | (41.4-61.8) |
| Damaged (65 - 642) | 0.0 | (--) | 4.8 | (2.6-8.7) | 15.8 | (9.3-25.7) | 28.3 | (19.5-39.2) |
| Torn (643+) | 0.0 | (--) | 3.5 | (1.7-7.1) | 15.8 | (7.7-29.9) | 20.0 | (11.9-31.7) |
| Serviceable (0 - 642) | 100.0 | (--) | 96.5 | (92.9-98.3) | 84.2 | (70.1-92.3) | 80.0 | (68.3-88.1) |
| Median pHI if any hole [IQR] | 24 | [1-26] | 25 | [4-86] | 52 | [5-580] | 144 | [28-584] |
| Has any repairs if any hole | 0.0 | (--) | 23.4 | (14.0-36.5) | 28.6 | (16.4-44.9) | 26.0 | (11.8-47.9) |
| **Gaoua (Interceptor®)** | **N=282** | | **N=206** | | **N=138** | | **N=94** | |
| Mean months since campaign | 4.3 | | 13.1 | | 24.1 | | 36.1 | |
| Net has any hole | 24.5 | (17.1-33.7) | 46.1 | (38.0-54.4) | 70.3 | (60.4-78.6) | 79.8 | (73.0-85.2) |
| **Physical condition (pHI)** |  |  |  |  |  |  |  |  |
| Good (0 - 64) | 94.0 | (94.5) | 90.3 | (85.5-93.6) | 63.8 | (56.0-70.9) | 54.3 | (43.8-64.3) |
| Damaged (65 - 642) | 5.0 | (3.2) | 6.8 | (4.1-11.2) | 24.6 | (19.1-31.1) | 25.5 | (18.2-34.6) |
| Torn (643+) | 1.1 | (2.3) | 2.9 | (1.2-6.7) | 11.6 | (7.2-18.2) | 20.2 | (11.9-32.2) |
| Serviceable (0 - 642) | 98.9 | (97.4) | 97.1 | (93.3-98.8) | 88.4 | (81.8-92.8) | 79.8 | (67.8-88.1) |
| Median pHI if any hole [IQR] | 24 | [2-62] | 16 | [2-49] | 69 | [24-341] | 99 | [24-662] |
| Has any repairs if any hole | 10.1 | (3.9) | 21.1 | (14.2-30.1) | 33.0 | (21.2-47.4) | 29.3 | (17.5-44.8) |
| **Orodara (PermaNet® 3.0)** | **N=346** | | **N=280** | | **N=209** | | **N=141** | |
| Mean months since campaign | 5.5 | | 14.4 | | 24.2 | | 36.1 | |
| Net has any hole | 29.5 | (21.0-39.6) | 65.4 | (54.9-74.5) | 81.8 | (85.5-93.3) | 90.1 | (85.5-93.3) |
| **Physical condition (pHI)** |  |  |  |  |  |  |  |  |
| Good (0 - 64) | 94.5 | (91.3-96.6) | 81.8 | (73.3-88.0) | 64.6 | (55.0-73.1) | 51.8 | (44.2-59.3) |
| Damaged (65 - 642) | 3.2 | (1.6-6.1) | 13.9 | (9.0-20.9) | 25.8 | (19.7-33.0) | 38.3 | (30.4-46.9) |
| Torn (643+) | 2.3 | (1.1-4.8) | 4.3 | (2.4-7.5) | 9.6 | (5.2-17.0) | 9.9 | (6.5-14.8) |
| Serviceable (0 - 642) | 97.4 | (94.3-98.8) | 95.7 | (92.5-97.6) | 90.4 | (83.0-94.8) | 90.1 | (85.2-93.5) |
| Median pHI if any hole [IQR] | 12 | [3-51] | 26 | [3-75] | 51 | [7-239] | 77 | [12-301] |
| Has any repairs if any hole | 3.9 | (1.2-12.2) | 13.7 | (9.7-18.9) | 44.4 | (33.0-56.5) | 56.7 | (43.7-68.8) |

*Table 6. Modelled Determinants of Physical Durability from Cox Proportional Hazard Models*

|  | **Adjusted HR** | **95% CI** | **p-value** |
| --- | --- | --- | --- |
| **Household-level model: 923 ITNs in 510 households** |  |  |  |
| Site [Ref: Banfora] |  |  |  |
| Gaoua | 1.27 | (0.88-1.81) | 0.198 |
| Orodara | 0.91 | (0.64-1.29) | 0.591 |
| Household with children under five-year-old [Ref: No children under five] |  |  |  |
| Yes with children under five | 1.21 | (0.94-1.56) | 0.148 |
| Household head sex [Ref: Male] |  |  |  |
| Female | 0.73 | (0.5-1.07) | 0.108 |
| Net SBC exposure [Ref: Never] |  |  |  |
| Exposed once | 0.69 | (0.5-0.95) | 0.022 |
| Exposed twice or more | 0.61 | (0.44-0.84) | 0.003 |
| Ever seen rodent [Ref: Always seen rodent] |  |  |  |
| Yes ever seen or had never seen rodent | 0.62 | (0.48-0.81) | 0.000 |
| **Net-level model: 719 ITNs ever hung in 424 households** |  |  |  |
| Site [Ref: Banfora] |  |  |  |
| Gaoua | 1.35 | (0.89-2.05) | 0.156 |
| Orodara | 0.58 | (0.35-0.94) | 0.026 |
| Ever seen rodent [Ref: Always seen rodent] |  |  |  |
| Yes ever seen or had never seen rodent | 0.66 | (0.50-0.88) | 0.005 |
| Household head sex [Ref: Male] |  |  |  |
| Female | 0.66 | (0.43-0.99) | 0.044 |
| Net washed with detergent [Ref: Not always washed with detergent] |  |  |  |
| Always | 1.36 | (0.93-1.99) | 0.113 |
| Type of sleeping place [Ref: Unfinished bed frame] |  |  |  |
| Finished bed frame | 1.31 | (0.94-1.83) | 0.113 |
| Net SBC exposure [Ref: Never] |  |  |  |
| Exposed once | 0.64 | (0.44-0.93) | 0.018 |
| Exposed twice or more | 0.52 | (0.36-0.74) | 0.000 |
| Nets folded [Ref: Never] |  |  |  |
| Folded once or more | 0.47 | (0.32-0.69) | 0.000 |
| Net care attitude [Ref: Never positive] |  |  |  |
| Positive once | 0.68 | (0.49-0.95) | 0.024 |
| Positive twice or more | 0.84 | (0.52-1.33) | 0.450 |
| HR: hazard ratio | | | |

*Table 7.* Characteristics of *strain*s used to test PermaNet® 3.0, Interceptor® and Interceptor® G2 ITNs

|  | **First round** | **Second round** | **Third round** | **Study endline** |
| --- | --- | --- | --- | --- |
| **Pyrethroid susceptible mosquito strain (*An. gambiae* Kisumu)** | | | | |
| % Mortality (24h) in deltamethrin (0.05%) susceptibility tests | 100% (n=90) | 100% (n=89) | 100% (n=96) | 100% (n=90) |
| % Mortality (24h) in alpha-cypermethrin susceptibility tests | 100% (n=95) | 100% (n=96) | 100% (n=95) | 100% (n=90) |
| % Mortality (24h) in deltamethrin (0.05%) + PBO (4%) synergist tests | n/a | 100% (n=89) | 100% (n=96) | 100% (n=96) |
| % Mortality (24h) in chlorfenapyr in CDC bottle bioassays at 100ug/bottle. | n/a | 100% (n=98) | 100% (n=95) | 100% (n=90) |
| Species composition of strain (PCR) | 100% *An. gambiae* (n=90) | 100% *An. gambiae* (n=92) | 100% *An. gambiae* (n=96) | 100% *An. gambiae* (n=90) |
| Kdr west (L1014F) frequency | f=0.0 (90 SS) (n=90) | f=0.0 (92 SS) (n=90) | f=0.0 (n=96) | f=0.0 (n=90) |
| Kdr east (L1014S) frequency | f=0.0 (90 SS) (n=90) | f=0.0 (92 SS) (n=90) | f=0.0 (n=96) | f=0.0 (n=90) |
| **Pyrethroid resistant mosquito strain (*An. coluzzii* VKPER)** | | | | |
| % Mortality (24h) in deltamethrin (0.05%) susceptibility tests | Test 1 33.0% (n=94)  Test 2 42.3% (n=104) | 39.4% (n=99) | 34.4% (n=90)) | 28.5% (n=90) |
| % Mortality (24h) in deltamethrin (0.05%) + PBO (4%) synergist tests | Test 1 89.6% (n=96)  Test 2 93.2% (n= 148) | 88.4% (n=105) | 91.8% (n=95) | 89.8% (n=90) |
| % Mortality (24h) in deltamethrin resistance intensity tests, 5X (0.25%) | 62.8% (n=94) | n/a | n/a | n/a |
| % Mortality (24h) in deltamethrin resistance intensity tests, 10X (0.50%) | 84.9% (n=106) | n/a | n/a | n/a |
| % Mortality (24h) in alpha-cypermethrin susceptibility tests | 13.3% (n=143) | 1.9% (n=107) | 6.4% (n=96) | 4.08% (n=90) |
| % Mortality (24h) in tunnel tests with new Interceptor® net (alpha-cypermethrin) | Test 1 12.0% (n=100)  Test 2 22.0% (n=91) | n/a | n/a | n/a |
| % Mortality (72h) in chlorfenapyr in CDC bottle bioassays at 100ug/bottle. | n/a | 95.7% (n=94) | 96.1% (n=98) | 94.8% (n=90) |
| % Mortality (72h) in chlorfenapyr in CDC bottle bioassays at 200ug/bottle. | n/a | 100% (n=99) | 100% (n=96) | 100% (n=90) |
| Species composition of strain (PCR) | 100% *An. coluzzii* (n=89) | 100% *An. coluzzii* (n=88) | 100% *An. coluzzii* | 100% *An. coluzzii* |
| Kdr west (L1014F) frequency | f=0.82 (58 RR, 30 RS, 1 SS) (n=89) | f=0.90 (69 RR, 16 RS) (n=85) | f=0.91 (71 RR, 14 RS) (n=85) | f=0.95 (81 RR, 9RS) (n=90) |
| Kdr east (L1014S) frequency | f=0.0 (89 SS) (n=89) | f=0.0 (85 SS) (n=85) | f=0.0 (85 SS) (n=85) | f=0.0 (90 SS) (n=90) |
| *n/a: Not available* | | | | |

*Table 8. Insecticide Effectiveness of Campaign ITNs*

|  | **Baseline** | | **Second round** | | **Third round** | | **Endline** | |
| --- | --- | --- | --- | --- | --- | --- | --- | --- |
|  | **% (95% CI)** | | **% (95 % CI)** | | **% (95 % CI)** | | **% (95 % CI)** | |
| **Banfora (Interceptor® G2)** | **N=30** | | **N=30** | | **N=24** | | **N=30** | |
| Mean months since campaign | 1.4 | | 10.1 | | 24.1 | | 33.1 | |
| **Tunnel test (Susceptible strain)** |  |  |  |  |  |  |  |  |
| Mortality 24 hours |  |  |  |  |  |  |  |  |
| Mean | 98.8 | (97.9-99.7) | 99.2 | (97.6-100) | 98.8 | (96.9-100) | 86.9 | (80.4-93.3) |
| Median [IQR] | 100 | (99.0-100) | 100 | (--) | 100 | (99.1-100) | 93.8 | (80.6-97.8) |
| Mortality 72 hours |  |  |  |  |  |  |  |  |
| Mean | 99.1 | (98.3-100) | 99.2 | (97.6-100) | 98.8 | (96.9-100) | 93.3 | (90.4-96.1) |
| Median [IQR] | 100 | (--) | 100 | (--) | 100 | (99.1-100) | 96.8 | (84.5-99.0) |
| Blood fed |  |  |  |  |  |  |  |  |
| Mean | 10.3 | (3.3-17.3) | 1.3 | (0.3-2.3) | 23.6 | (14.3-32.9) | 21.0 | (14.7-27.3) |
| Median [IQR] | 2.8 | (0.9-12.8) | 0.6 | (0.0-1.2) | 19.8 | (6.8-40.8) | 18.5 | (7.0-32.6) |
| Blood-feeding inhibition |  |  |  |  |  |  |  |  |
| Mean | 89.2 | (81.6-96.7) | 97.6 | (95.4-99.7) | 50.9 | (31.4-70.3) | 78.8 | (72.4-85.2) |
| Median [IQR] | 97.1 | (85.7-99.1) | 99.0 | (97.7-100) | 58.9 | (15.0-85.8) | 81.1 | (67.6-93.0) |
| **Tunnel test (Resistant strain)** |  |  |  |  |  |  |  |  |
| Mortality 24 hours |  |  |  |  |  |  |  |  |
| Mean | 71.2 | (65.0-77.3) | 55.1 | (48.2-61.9) | 57.3 | (44.1-70.5) | 44.5 | (32.4-56.6) |
| Median [IQR] | 72.5 | (63.7-82.9) | 57.0 | (43.8-64.6) | 66.5 | (39.1-77.2) | 43.6 | (15.9-68.6) |
| Mortality 72 hours |  |  |  |  |  |  |  |  |
| Mean | 84.5 | (79.7-89.3) | 66.2 | (58.7-73.6) | 71.8 | (57.9-85.6) | 50.5 | (39.5-61.4) |
| Median [IQR] | 86.7 | (75.0-92.5) | 70.2 | (57.1-80.0) | 78.7 | (54.2-94.3) | 49.6 | (25.6-71.7) |
| Blood fed |  |  |  |  |  |  |  |  |
| Mean | 43.0 | (35.2-50.8) | 41.7 | (34.7-48.6) | 35.2 | (24.9-45.4) | 39.3 | (27.7-50.9) |
| Median [IQR] | 42.0 | (26.0-59.5) | 41.2 | (25.6-54.3) | 35.4 | (23.4-47.3) | 39.2 | (15.2-50.9) |
| Blood-feeding inhibition |  |  |  |  |  |  |  |  |
| Mean | 55.2 | (47.1-63.3) | 55.1 | (48.1-62.1) | 61.9 | (50.8-73.1) | 53.6 | (38.4-68.8) |
| Median [IQR] | 52.8 | (37.4-74.0) | 55.3 | (39.6-71.8) | 61.7 | (48.8-74.7) | 51.4 | (38.2-84.6) |
| **Chemical Content** |  |  |  |  |  |  |  |  |
| Alpha-cypermethrin |  |  |  |  |  |  |  |  |
| Mean | 2.7 | (2.6-2.8) | 2.3 | (2.2-2.5) | 2.0 | (1.6-2.3) | 1.7 | (1.4-2.0) |
| Median [IQR] | 2.7 | (2.6-2.8) | 2.4 | (2.2-2.7) | 2.2 | (1.5-2.5) | 1.8 | (1.0-2.3) |
| Chlorfenapyr |  |  |  |  |  |  |  |  |
| Mean | 5.0 | (4.7-5.3) | 3.5 | (3.2-3.9) | 2.2 | (1.6-2.9) | 1.6 | (1.1-2.1) |
| Median [IQR] | 5.0 | (4.6-5.3) | 3.9 | (3.0-4.6) | 2.2 | (1.0-3.2) | 1.2 | (0.1-2.8) |
| **Gaoua (Interceptor®)** | **N=30** | | **N=30** | | **N=30** | | **N=30** | |
| Mean months since campaign | 4.3 | | 13.1 | | 24.1 | | 36.1 | |
| **Cone bioassays** |  |  |  |  |  |  |  |  |
| Knock down 60 minutes |  |  |  |  |  |  |  |  |
| Mean | 96.9 | (95.9-98.0) | 94.0 | (89.5-98.6) | 95.8 | (90.3-100) | 75.8 | (65.6-86.1) |
| Median [IQR] | 97.7 | (95.9-100) | 97.7 | (93.5-100) | 100 | (97.4-100) | 80.8 | (61.4-94.0) |
| Mortality 24 hours |  |  |  |  |  |  |  |  |
| Mean | 99.9 | (99.7-100) | 99.2 | (98.2-100) | 99.5 | (98.8-100) | 70.8 | (58.0-83.7) |
| Median [IQR] | 100 | (--) | 100 | (--) | 100 | (--) | 78.8 | (43.5-96.0) |
| Optimal effectiveness | 100 | (--) | 100 | (--) | 100 | (--) | 60.0 | (37.2-82.8) |
| Minimal effectiveness | 100 | (--) | 100 | (--) | 100 | (--) | 86.7 | (70.4-100) |
| **Chemical Content** |  |  |  |  |  |  |  |  |
| Mean | 3.3 | (2.7-4.0) | 2.7 | (1.9-3.6) | 1.7 | (1.2-2.2) | 0.8 | (0.3-1.3) |
| Median [IQR] | 3.3 | (2.0-4.5) | 2.5 | (0.9-4.5) | 1.3 | (0.6-2.1) | 0.5 | (0.1-1.2) |
| **Orodara (PermaNet® 3.0)** | **N=30** | | **N=30** | | **N=30** | | **N=30** | |
| Mean months since campaign | 5.5 | | 14.4 | | 24.2 | | 36.1 | |
| **Cone bioassays (Susceptible strain)** |  |  |  |  |  |  |  |  |
| Sides (pyrethroid-only) |  |  |  |  |  |  |  |  |
| Knock down 60 minutes |  |  |  |  |  |  |  |  |
| Mean | 97.2 | (96.0-98.5) | 98.9 | (97.9-100) | 94.3 | (86.5-100) | 84.9 | (78.1-91.8) |
| Median [IQR] | 98.8 | (96.6-100) | 100 | (--) | 100 | (--) | 91.6 | (80.0-97.1) |
| Mortality 24 hours |  |  |  |  |  |  |  |  |
| Mean | 100 | (--) | 99.1 | (97.4-100) | 97.2 | (93.8-100) | 71.9 | (61.3-82.5) |
| Median [IQR] | 100 | (--) | 100 | (--) | 100 | (--) | 83.7 | (54.8-100) |
| Roof (pyrethroid + PBO) |  |  |  |  |  |  |  |  |
| Knock down 60 minutes |  |  |  |  |  |  |  |  |
| Mean | 100 | (93.0-100) | 99.3 | (98.9-99.7) | 97.2 | (92.8-100) | 95.5 | (91.4-99.6) |
| Median [IQR] | 100 | (93.3-100) | 100 | (--) | 100 | (--) | 98.7 | (94.3-100) |
| Mortality 24 hours |  |  |  |  |  |  |  |  |
| Mean | 100 | (--) | 99.9 | (99.7-100) | 95.4 | (88.1-100) | 95.4 | (90.2-100) |
| Median [IQR] | 100 | (--) | 100 | (--) | 100 | (--) | 100 | (100-100) |
| **Cone bioassays (Resistant strain)** |  |  |  |  |  |  |  |  |
| Sides (pyrethroid-only) |  |  |  |  |  |  |  |  |
| Knock down 60 minutes |  |  |  |  |  |  |  |  |
| Mean | 42.4 | (34.0-50.8) | 42.1 | (28.1-56.0) | 34.1 | (23.5-44.6) | 17.2 | (11.8-22.6) |
| Median [IQR] | 42.5 | (22.2-60.6) | 36.5 | (19.4-69.2) | 24.8 | (11.9-48.0) | 11.6 | (6.1-21.9) |
| Mortality 24 hours |  |  |  |  |  |  |  |  |
| Mean | 23.8 | (18.6-28.9) | 52.6 | (43.4-61.8) | 21.1 | (9.6-32.6) | 10.9 | (6.6-15.1) |
| Median [IQR] | 20.9 | (12.9-30.6) | 54.3 | (25.7-74.1) | 13.1 | (4.1-25.6) | 7.1 | (2.9-11.4) |
| Roof (pyrethroid + PBO) |  |  |  |  |  |  |  |  |
| Knock down 60 minutes |  |  |  |  |  |  |  |  |
| Mean | 85.2 | (76.9-93.4) | 67.9 | (57.7-78.1) | 69.7 | (57.4-82.0) | 44.5 | (34.5-54.4) |
| Median [IQR] | 90.0 | (83.9-96.7) | 74.6 | (54.3-85.2) | 77.4 | (60.9-90.7) | 36.0 | (25.0-60.6) |
| Mortality 24 hours |  |  |  |  |  |  |  |  |
| Mean | 71.7 | (64.0-79.5) | 75.1 | (69.2-81.0) | 51.6 | (38.6-64.5) | 25.7 | (15.8-35.5) |
| Median [IQR] | 69.8 | (58.1-87.5) | 79.7 | (61.3-90.3) | 48.8 | (32.6-73.2) | 16.6 | (8.3-38.2) |
| **Chemical Content** |  |  |  |  |  |  |  |  |
| Pyrethroid - side panels |  |  |  |  |  |  |  |  |
| Mean | 1.5 | (1.2-1.7) | 0.8 | (0.6-1.1) | 0.8 | (0.4-1.1) | 0.3 | (0.1-0.4) |
| Median [IQR] | 1.7 | (0.9-1.9) | 0.6 | (0.4-1.4) | 0.4 | (0.3-1.4) | 0.1 | (0.0-0.3) |
| Pyrethroid - roof |  |  |  |  |  |  |  |  |
| Mean | 3.5 | (3.4-3.7) | 3.3 | (3.1-3.4) | 3.0 | (2.8-3.2) | 2.6 | (2.4-2.9) |
| Median [IQR] | 3.6 | (3.3-3.8) | 3.4 | (3.0-3.5) | 3.0 | (2.7-3.3) | 2.8 | (2.5-2.9) |
| PBO - roof |  |  |  |  |  |  |  |  |
| Mean | 15.2 | (13.5-16.9) | 9.0 | (7.4-10.7) | 7.8 | (5.9-9.8) | 3.9 | (3.0-4.8) |
| Median [IQR] | 14.5 | (12.1-17.2) | 8.2 | (6.2-9.8) | 6.7 | (5.1-10.2) | 3.4 | (2.6-4.4) |
